# Activation and Allostery in a Fungal SAMHD1 Hydrolase: An Evolutionary Blueprint for dNTP Catabolism

**DOI:** 10.1021/jacsau.5c00090

**Published:** 2025-04-17

**Authors:** Luying Pan, Jake C. Lachowicz, Isaac Paddy, Yutong Xu, Qianyi Yang, Cynthia Zizola, Amy Milne, Tyler L. Grove, Maria-Eirini Pandelia

**Affiliations:** † Department of Biochemistry, Brandeis University, Waltham, Massachusetts 02453, United States; ‡ Department of Biochemistry, Albert Einstein College of Medicine, Bronx, New York 10461, United States

**Keywords:** HD-domain, iron, manganese, allostery, Rhizophagus irregularis

## Abstract

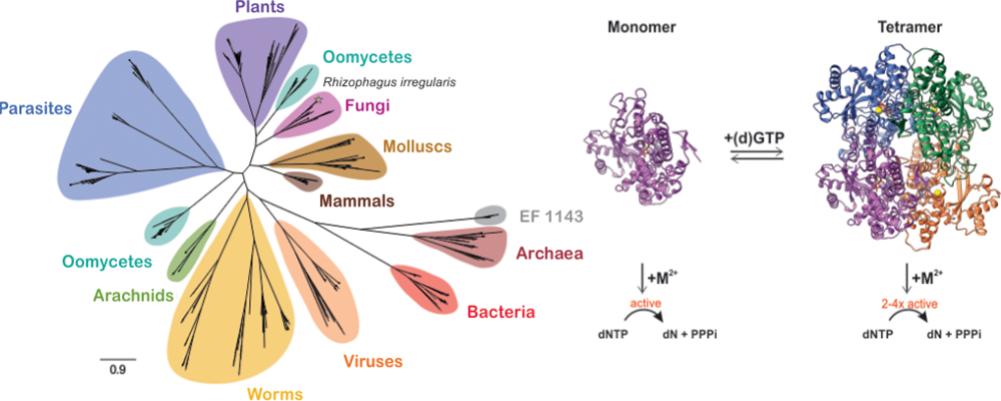

Sterile alpha motif and HD domain-containing protein 1 (SAMHD1) is a metal-dependent hydrolase that plays key roles in dNTP homeostasis, antiretroviral defense, and regulation of various cancers in humans. Beyond mammals, SAMHD1 is also present in a wide range of eukaryotes, including invertebrates, plants, and human parasites. Although the specific mechanisms and biological significance of SAMHD1 in these organisms are not well understood, its functions are linked to essential processes such as photosynthesis, genome maintenance, and immune response. In this study, we bioinformatically mined the SAMHD1 superfamily and selected the ortholog from the mycorrhizal fungus *Rhizophagus irregularis* as a model system for both fungal and biochemically intractable plant SAMHD1s. *Ri* SAMHD1 retains the substrate promiscuity of the human enzyme but bypasses the strict requirement for allosteric activation through tetramerization, positioning it as a prototypical enzyme in which hydrolysis and allosteric regulation can be uncoupled. Its activity is selectively dependent on transition metal ions such as Mn and Fe, while Mg serves as a poor activator. Although *Ri* SAMHD1 lacks several ancillary regulatory features present in human SAMHD1, its activity is differentially modulated by GTP, which acts as an allosteric activator at lower concentrations and an allosteric inhibitor at higher concentrations. These results demonstrate that metal dependence and allosteric regulation are adaptive traits that have evolved divergently among mammals, fungi, and plants, invoking alternative molecular routes for fine-tuning dNTP levels. Our findings on *Ri* SAMHD1 provide a paradigm for the mechanistic diversification of SAMHD1 enzymes and offer valuable insights for dissecting the complex mechanisms of nucleotide regulation in humans.

## Introduction

Sterile alpha motif and HD domain-containing protein 1 (SAMHD1) is a metal-dependent hydrolase that catalyzes the breakdown of cellular dNTPs, thereby regulating dNTP levels in humans, mice, and other mammals.^1−7^ SAMHD1 has been well-recognized as an antiviral factor against HIV-1, depleting the cellular pool of dNTPs necessary for viral DNA synthesis.^8,9^ Activity of SAMHD1 extends beyond its antiviral role; it is also involved in diverse cellular processes, including double-strand break repair, genome stability, immune response, and replication stress response via interferon signaling.^10−14^ SAMHD1 mutations are associated with the Aicardi-Goutières syndrome (AGS), a rare genetically determined encephalopathy characterized by increased interferon expression, resembling viral infection.^15,16^ In humans, SAMHD1 downregulation or mutations have also been identified in various cancer types, invoking a correlation between SAMHD1 repression and cancer development.^17,18^ In this respect, genomic instability and cancer development associated with SAMHD1 mutations may arise from either compromised dNTPase activity or other SAMHD1-mediated processes, such as defective DNA end resection at stalled replication forks.^1,3,11,12^

SAMHD1 is found not only in mammals but also in a diverse range of eukaryotes, including invertebrates, plants, and human parasites.^19−25^ In the nematode *Caenorhabditis elegans*, reduced expression of the SAMHD1 protein ZK177.8 results in maternal sterility.^26^ In rice (*Oryza sativa*), mutations in *samhd1* impair photosynthetic capacity by disrupting chlorophyll biosynthesis and chloroplast ribosome biogenesis.^19,20^ Similarly, *samhd1* mutations in cucumber (*Cucumis sativus*) and thale cress (*Arabidopsis thaliana*) lead to diminished leaf coloration and improper chloroplast development.^21,22^ Furthermore, *At* SAMHD1 is essential for plant immunity and acclimation to adverse environmental conditions.^21,23^ On the other hand, in the human parasite *Trypanosoma brucei*, the causative agent of sleeping sickness, deletion of *samhd1* leads to defects in kinetoplast and genomic integrity.^24,25^ Although SAMHD1 is widespread across these diverse organisms, the specific functional roles and cellular pathways mediated by its orthologs are still poorly understood.

Mammalian SAMHD1 comprises a SAM domain (residues 45–110 in *Hs* SAMHD1) and a histidine-aspartic (HD) domain (residues 112–582 in *Hs* SAMHD1) that harbors the active and allosteric sites essential for hydrolytic activity.^27,28^ In humans, the SAM domain has minimal influence on dNTP hydrolysis, whereas in mice, it is essential for allosteric activation.^27,29^ Until 2020, SAMHD1 was thought to bind a single metal ion at its active and allosteric sites, respectively. This ion was identified as either Fe or Zn in several resolved crystal structures, while activity was stimulated by the addition of either Mg or Mn.^1,30−33^ Structural studies by Morris et al. uncovered a heteronuclear Fe/Mg active site and proposed that this represents the biologically relevant cofactor form.^34−36^ A third Mg ion was present, but coordinated by the phosphate oxygens without directly interacting with the protein. The Mg at the active site could be substituted with Mn, demonstrating that Mg is labile, whereas Fe is stably coordinated.^34^ These observations led to a two-metal-based mechanism for dNTP hydrolysis and implied that SAMHD1 functions as an Fe-dependent hydrolase, although the precise role of Fe in activity remained cryptic.

SAMHD1 activity is tightly regulated through tetramerization, which is dependent on the binding of nucleotides and metal ions to two allosteric sites, AL1 and AL2.^36,37^ Clinical mutations in residues at the AL1, AL2, and oligomeric interfaces significantly reduce or abolish dNTP hydrolysis.^1,30,31,38^ When isolated, the protein predominantly exists as a monomer that lacks any dNTPase activity.^1,37^ Binding of a metal-GTP effector to AL1 induces formation of an inactive dimer.^27,31,37,39,40^ A second dNTP binds to form a metal-centered dinucleotide between AL1 and AL2, resulting in the tetrameric form potent for catalysis.^27,31,33,37,39−41^ Activity of *Hs* SAMHD1 is additionally regulated by various mechanisms, further highlighting the necessity for a fine-tuned control of its function. Cysteines C341, C350, and C522 are involved in intramolecular disulfide bonds that reversibly inhibit tetramerization and catalysis, acting as a redox switch to regulate hydrolysis and promote ssDNA binding during the S-phase.^42−44^ Moreover, SAMHD1 is phosphorylated at the C-terminal threonine T592 by cyclin-dependent kinases, impairing its ability to block retroviral infection via a mechanism that is independent of dNTP hydrolysis.^45^ Furthermore, binding of nucleic acids results in mixed-occupancy tetramers, altering the retroviral activity of SAMHD1 without affecting its dNTPase function.^36,46^

*Rhizophagus irregularis* is a mycorrhizal fungus that colonizes plant roots, facilitating the transfer and uptake of mineral nutrients from the soil to plants. This property makes it a common component in mycorrhizal-based fertilizers.^47^*Ri* significantly enhances the yield and nutritional value of various plant species, particularly under abiotic stress conditions such as nutrient deficiency, drought, and heavy metal contamination.^48−52^ Despite its well-established role in plant health, the need for or biological significance of the SAMHD1 protein encoded in *Ri* remains unclear. Given homology based on sequence similarity (53% with *Hs* SAMHD1, 49% with rice (*Os*) SAMHD1, and 53% with cucumber (*Cs*) SAMHD1), *Ri* SAMHD1 was selected as a model system to explore the mechanisms of dNTP hydrolysis in mutualistic soil fungi and plants. Interestingly, dG derived from endophytic fungal metabolites has been shown to trigger an immune response in plants, although the exact molecular basis of this effect remains unclear.^23^

In this study, we present the first three-dimensional structure of the fungal *Ri* SAMHD1 and detail its activity profile, active site chemical makeup, and regulatory activation/inhibition mechanisms. While *Ri* SAMHD1 shares many catalytic and regulatory features with *Hs* SAMHD1, it also exhibits unique features in the allosteric mechanisms that can be both activating as well as inhibitory. Notably, *Ri* SAMHD1 is active in its monomeric form, making it the first known SAMHD1 model in which hydrolysis can be decoupled from allosteric regulation. This study offers valuable insights into the diverse mechanisms that nature employs to regulate dNTP pools, positioning *Ri* SAMHD1 as an archetypal model for addressing previously unresolved questions about SAMHD1 activation and catalysis in humans and plants.

## Materials and Methods

### Materials

All chemicals unless specified were obtained from Fisher Scientific (NH) and were of high purity grade. dNTPs were purchased by Thermo Scientific (MA) and G-Biosciences (MO), while NTPs were purchased by Cytiva (MA) and Thermo Scientific (MA).

### Sequence Similarity Network (SSN)

A BLAST search was performed using the sequence of the human SAMHD1 (NCBI Reference Sequence: NP_056289.2) against proteins in the RefSeq database. The SSN was generated with the web-based Enzyme Function Initiative-Enzyme Similarity Tool (EFI-EST) and visualized in Cytoscape.^53,54^ The network was further refined by employing an alignment score of 120 and restricting the length of the obtained sequences to 300–750 amino acids to exclude truncated or partial sequences. The final optimized SSN contains 4,466 unique sequences and 3,428 nodes, with each node representing proteins that share at least 95% sequence identity.

### Phylogenetic Analysis

We selected 451 unique and nonredundant sequences from the major protein clusters of the SAMHD1 SSN that represent the bulk of the organisms in which SAMHD1 occurs. The sequences were aligned with the MAFFT software (https://mafft.cbrc.jp/alignment/software/)^55^ and the maximum-likelihood phylogenetic rooted tree was subsequently computed with the IQ-tree software (http://www.iqtree.org) employing LG + R10 model.^56^ 14 sequences of the distant homologue HD-domain protein EF1143 were used as the outgroup.^57^

### Plasmid Construction and Mutagenesis

The wild-type (WT) encoding gene of the SAMHD1 protein from *Rhizophagus irregularis* (NCBI Reference Sequence: XP_025183678.1) was synthesized and cloned into a pET28a(+) expression vector (GenScript, NY) that allows for its expression with an N-terminal His_6_-tag. The D34A variant was generated by GenScript Inc. The H129A and D103R/H104N variants were generated using the primers listed in Table S1 by back-to-back PCR (Q5 Mutagenesis, New England Biolabs, MA). All sequences were confirmed by Sanger sequencing (Azenta Inc., NY).

### Protein Expression and Purification

All plasmids were transformed into T7 Express *Escherichia coli* competent cells (New England Biolabs, MA) and selected for kanamycin resistance. To direct specific metal incorporation, transformed cells were grown in minimal (M9). Details about the expression and purification of *Ri* SAMHD1 can be found in the Supporting Information.

### Crystallization

Diffraction-quality crystals were obtained by sitting-drop vapor diffusion at 20 °C in an anaerobic chamber maintained at <0.1 ppm oxygen (MBraun). Drops of 0.4 μL of *Ri* SAMHD1 (10 mg/mL *Ri* SAMHD1, 2.5 mM MnCl_2_, and 5 mM GTP) were mixed with 0.4 μL of precipitant (0.2 M ammonium tartrate, 20% (w/v) PEG 3350) and equilibrated against a solution of 0.5 M LiCl. Crystals were mounted in nylon loops and flash-cooled in liquid nitrogen inside the anaerobic chamber and were stored in liquid nitrogen prior to data collection. Diffraction data were collected at NSLSII (Brookhaven National Lab, Brookhaven, NY) on beamline 17-ID-2 (FMX). All diffraction data were integrated and scaled using the HKL3000 suite or XDS and aimless. Diffraction data was consistent with the triclinic space group *P 1* and a resolution of 2.27 Å with unit cell parameters consisting of *a* = 104.58 Å, *b* = 111.635 Å, and *c* = 99.513 Å (*a* = 84.359°, *b =* 85.42°, *c* = 87.035°*)* with four molecules per asymmetric unit. Phases were determined by molecular replacement using the previously solved structure of the tetrameric human D137N-SAMHD1 (PDB: 6XT0). Molecular replacement was performed using PHENIX,^58^ with manual model building using Coot,^59^ and refinement using phenix.refine.^58^ The final model of *Ri* SAMHD1 consists of four molecules in the asymmetric unit with chains consisting of residues 11–451. K373 of chain A and N388 of chain B are not modeled due to poor density. Each monomer contained one calcium ion, one manganese ion, and one molecule of GTP within an asymmetric unit. There is weak density within the active site consistent with a nucleotide triphosphate, with stronger density adjacent to the manganese ion likely corresponding to pyrophosphate. This density is likely the breakdown product from GTP and, therefore, only pyrophosphate was modeled in the active site. There was also additional density within the allosteric site, adjacent to the calcium ion, suggesting a nucleotide also exists within the allosteric site similar to other published structures of SAMHD1. However, this density was too weak to model. All figures were produced using PyMOL (Schrödinger, LLC) and ChimeraX (https://www.rbvi.ucsf.edu/chimerax). Data collection and refinement statistics are shown in Table S2.

### Elemental Analysis

Metal analysis of the protein samples was determined by inductively coupled atomic emission spectrometry (ICP-AES) at the Environmental Sustainability Laboratories (EESL) at the Pennsylvania State University (PA). Samples were prepared by precipitation in 3.5% metal-free nitric acid.

### Electron Paramagnetic Resonance (EPR) Spectroscopy

All samples were prepared in storage buffer (50 mM MES, 150 mM NaCl, 50 mM l-arginine HCl, 10% glycerol, pH 6.5) under aerobic conditions or O_2_-free conditions in an anaerobic glovebox. The as-isolated samples were reduced with 2 mol equivalents (eq) sodium ascorbate for 10 min at room temperature prior to freezing in liquid N_2_. The nucleotide-added samples were reduced with 2 molar eq sodium ascorbate for 10 min at room temperature prior to addition of GTP/dNTP and freezing in liquid N_2_. EPR spectra were acquired on a Bruker E500 Elexsys continuous wave (CW) X-Band spectrometer (operating at approximately 9.36 GHz) equipped with a rectangular resonator (TE102) and a continuous-flow cryostat (Oxford 910) with a temperature controller (Oxford ITC 503).

### Mössbauer Spectroscopy

All samples were prepared in the storage buffer (50 mM MES, 150 mM NaCl, 50 mM l-arginine HCl, 10% glycerol, pH 6.5) under aerobic conditions or O_2_-free conditions in an anaerobic glovebox (CoyLab). Samples were reacted with 10 mM sodium dithionite for 15 min to allow for accumulation of the Fe_2_^II^ state. Mössbauer spectra were recorded on WEB Research (Edina, MN) instrument.^60^ The spectrometer used to acquire the weak-field spectra is equipped with a Janis SVT-400 variable-temperature cryostat. The external magnetic field was applied parallel to the γ beam. All isomer shifts are quoted relative to the centroid of the spectrum of α-iron metal at room temperature. Mössbauer spectra were fitted using the WMOSS4 Mössbauer Spectral Analysis Software (www.wmoss.org).

### High Performance Liquid Chromatography (HPLC)

Denatured reaction samples were centrifuged at 21,130*g* and the supernatant was filtered with 0.22 μm nylon Spin-X Centrifuge Filter (Corning Incorporated, NY). The filtered samples were analyzed on a 1260 Infinity Liquid Chromatography system (Agilent, CA) and a 1260 Infinity Photodiode Array Detector WR by monitoring the absorbance at 254 nm. Samples were injected on an Agilent reverse-phase C18- A Polaris column (particle size 5 μm, 150 × 4.6 mm). Educts and products were separated using a gradient method utilizing a water-based mobile phase (10 mM KH_2_PO_4_ and 10 mM tetrabutylammonium hydroxide (TBAH), pH 6, Solvent A) and an organic-based mobile phase (methanol with 10 mM TBAH, Solvent B). Quantification was afforded by the relative integrated peak intensities of educts and products.

### Chemical Cross-Linking

The cross-linking reactions were prepared in the assay buffer (50 mM MES hydrate, 150 mM NaCl, pH 6.5) in which the final protein concentration was 2 μM. The samples were first incubated with a series of different transition metal ion salts Fe^2+^ (0.5 mM (NH_4_)_2_Fe(SO_4_)_2_), Mn^2+^ (1 mM MnCl_2_), Mg^2+^ (1 mM MgCl_2_), and Co^2+^ (1 mM CoCl_2_) and the respective nucleotides (ATP, UTP, CTP, GTP, dATP, dTTP, dCTP, and dGTP) for 15 min, and then treated with an equal volume of 20 mM glutaraldehyde for 15 min. The as-isolated samples were treated with an equal volume of 20 mM glutaraldehyde for 15 min. The reactions were quenched by addition of 8 μL of 1 M tris(hydroxymethyl)aminomethane hydrochloride (Tris-HCl) (pH 7.5). The samples were treated with 16 μL 4× SDS loading dye and loaded onto 8% SDS-PAGE gel to resolve the bands. The gels were imaged on a ChemiDoc Imager (Bio-Rad) and band intensities were quantified using the software ImageJ.^61^

## Results

### Structure of *Ri* SAMHD1

**Figure 1:**
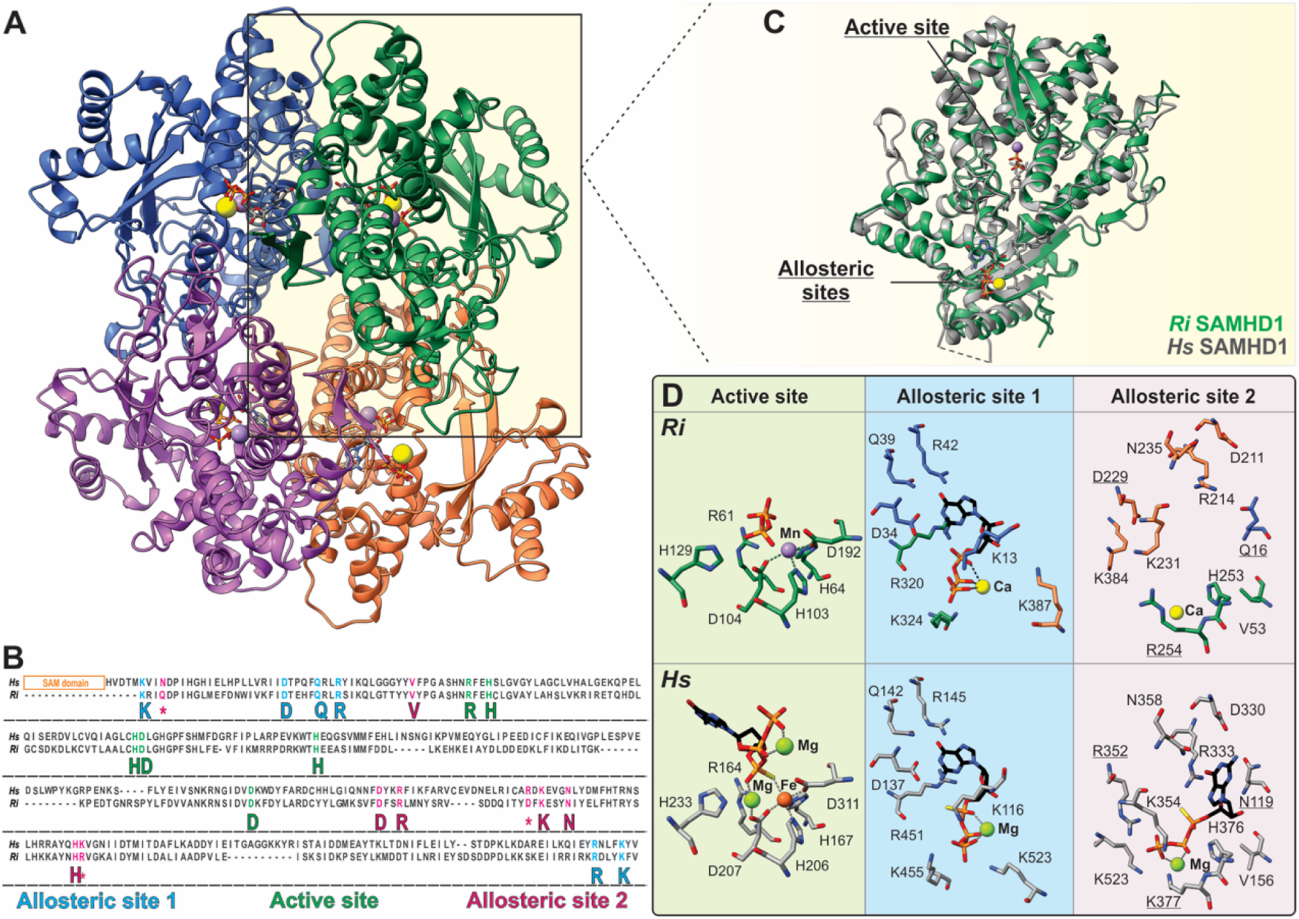
Structure and sequence comparison of *Ri* and *Hs* SAMHD1. (A) Three-dimensional structure of the tetrameric form of M9-Mn *Ri* SAMHD1 obtained after cocrystallization with GTP and excess Mn (PDB: 9MR6). The four subunits are shown as ribbons and colored differently on the basis of their subunit composition. Subunits A, B, C, and D are colored in blue, green, purple, and orange, respectively. GTP is shown as sticks, Mn^2+^ as purple, and Ca^2+^ as yellow sphere. (B) Sequence alignment between *Ri* and *Hs* SAMHD1. AL1 (blue), AL2 (magenta), catalytic site (green), and SAM domain (orange) are highlighted. AL2 residues that differ between sequences are indicated by asterisks. (C) Overlay of *Ri* SAMHD1 (green, PDB: 9MR6) and *Hs* SAMHD1 (gray, PDB: 7A5Y), both shown in cartoon representation. (D) Detailed views of the active sites and allosteric sites of *Ri* SAMHD1 (green, PDB: 9MR6) and *Hs* SAMHD1 (gray, PDB: 7A5Y). The *Ri* SAMHD1 residues are colored according to each subunit and correspond to the subunit color in the tetramer. Bound nucleotides are colored in black. Coordination bonds between metal ions and amino acids are shown as dotted lines. The metal ions Mn^2+^, Mg^2+^, Ca^2+^, and Fe^2+^ are represented by purple, green, yellow, and orange spheres, respectively.

*Ri* SAMHD1 was expressed under various metal supplementation conditions, and only the protein produced from minimal media promoting Mn incorporation yielded well-diffracting crystals. The 2.27 Å resolution structure of *Ri* SAMHD1 in the presence of excess Mn and GTP as a nucleotide effector (PDB code: 9MR6) shows that, under these conditions, the protein forms a tetramer in the asymmetric unit (Figure 1A). A tetrameric form has also been observed for the mammalian (human, mouse, pig) and roundworm *Ce* SAMHD1, for all of which this represents the biologically relevant form.^29,37,62,63^ The symmetry of the tetramer resembles that observed for *Hs* SAMHD1 demonstrating similar assembly and subunit contacts (Figure S1). While a member of the SAMHD1 subfamily, *Ri* SAMHD1 lacks the N-terminal SAM domain (Figures 1B, S2), a feature shared with other nonmammalian homologues (*vide infra*). *Ri* and *Hs* SAMHD1 share 53% sequence identity (Table S3) and are structurally homologous with a root-mean-square deviation of 0.981 Å (Figure S3). Despite the appreciable sequence and structural similarity, there are some differences between the two structures that may be of functional significance. *Ri* lacks a loop (residues 276–279) in the vicinity of the dimer interface, has a loop instead of a helix (residues 148–152) near the allosteric sites, and exhibits a shortened loop (residues 166–175) and β-sheet (residues 223–228) on the surface (Figures 1C, S3).

#### Active Site

The active site contains a single Mn ion coordinated by four residues, including the HD dyad (i.e., H64, H103, D104, and D192), a configuration identical to that of the Fe-bound structures of *Hs* SAMHD1, whether mononuclear or dinuclear (Figures 1B, D, S4, S5).^30,34−36^ Although *Ri* lacks a second metal ion, H129 – the counterpart of H233, which coordinates the Mg ion in the Fe–Mg site of Hs SAMHD1 – is similarly positioned in terms of distance and orientation (Figure 1B, D).^34^ These results are suggestive of the presence of a bimetallic site in *Ri* SAMHD1, with the second metal ion not observed under our crystallization conditions (Figure 1D). While a second Mn ion was not detectable in our structure, an additional weak electron density was present near the Mn ion, that was best fitted with a pyrophosphate molecule. R61 in *Ri* SAMHD1 is the cognate residue of R164 in *Hs* SAMHD1, a strictly conserved arginine that forms a salt bridge with the dNTP phosphate and is essential for hydrolytic activity.^1^ The presence and similar orientation of R61 demonstrate that this arginine is a characteristic feature of SAMHD1 dNTPases specifically, and HD-domain phosphatases more broadly (Figure 1B, D).^1,30,64^

#### Allosteric Sites

In the *Ri* SAMHD1 tetramer, only one nucleotide (GTP) is positioned within the interface of three subunits (A, B, and D), forming an extensive network of hydrogen-bonding interactions with residues from the two allosteric sites (Figure 1D). The GTP primarily interacts with D34, Q39, and R42 of subunit A (AL1), forming five hydrogen-bonding interactions with the Watson–Crick and the G base Hoogsteen (N7) sites. Additional hydrogen-bonding interactions with the triphosphate group are provided by residues from subunits A (K13), B (R320, K455), and D (K387), all of which are similarly positioned to those in *Hs* SAMHD1 (Figure 1D). The GTP is coordinated via its phosphate oxygens in a tridentate fashion to a metal ion, which, on the basis of the absence of any anomalous scattering, cannot be assigned to Mn (added in excess) or another transition metal. Elemental analyses show that the enzyme copurifies with approximately 3.5 eq of Ca^2+^ and only trace amounts of Mg (0.1 eq). Given that the coordination geometry deviates from the typical octahedral arrangement found in Mg ions and coupled with the minimal presence of Mg in our samples, we have assigned this metal to a calcium ion.

The orientation of the AL2 residues is similar to that observed in the dGTP-bound structure of *Hs* SAMHD1 (Figure 1D).^27,31,33,35^ Despite the absence of a second nucleotide in *Ri* SAMHD1, D211 and N235 from subunit D adopt similar conformations to D330 and N358 in *Hs* SAMHD1, respectively. The residues that form hydrogen bonds with the triphosphate group of dGTP, such as H253 (subunit B), and R214, K231, and K387 (subunit D), are also conserved and similarly positioned to those in the human enzyme. In addition, there is some conservation in the sugar interactions between *Ri* and *Hs* SAMHD1, such as in subunit A (Q16 vs N119), B (V53 vs V156), and D (R214 vs R333). Despite the similarities, there are some differences in AL2 such as K377 (subunit B) and R352 (subunit D) in *Hs* SAMHD1 are replaced by R254 and D229 in *Ri* SAMHD1 (Figure 1D). Overall, the allosteric sites are arranged similarly to those in the human enzyme, with the most notable differences being the presence of a calcium ion and the absence of a second nucleotide effector.

#### Regulatory Sites

*Ri* SAMHD1 lacks threonine T592, which is phosphorylated in the human enzyme, as well as the three cysteines that function as a redox switch (Figure S2).^42,44,45^ Absence of these regulatory sites suggests that activity regulation in *Ri* in vivo is likely simpler and less controlled than that in *Hs* SAMHD1 or may occur through alternative mechanisms. In this context, *Ri* SAMHD1 exhibits some differences in the two hydrophobic patches that play a role in stabilizing the dimer interface.^65^ The crucial for dimerization residues Y146, Y154, L428, and Y432 in *Hs* SAMHD1 are replaced by residues S43, Y51, N295, and Y299 in *Ri* SAMHD1 (Figures S2, S6). Despite these substitutions, *Ri* SAMHD1 is still able to oligomerize, suggesting that changes in hydrophobicity and charge do not adversely impact its dimerization.

### Chemical Nature of the *Ri* SAMHD1 Metallocofactor

**Figure 2:**
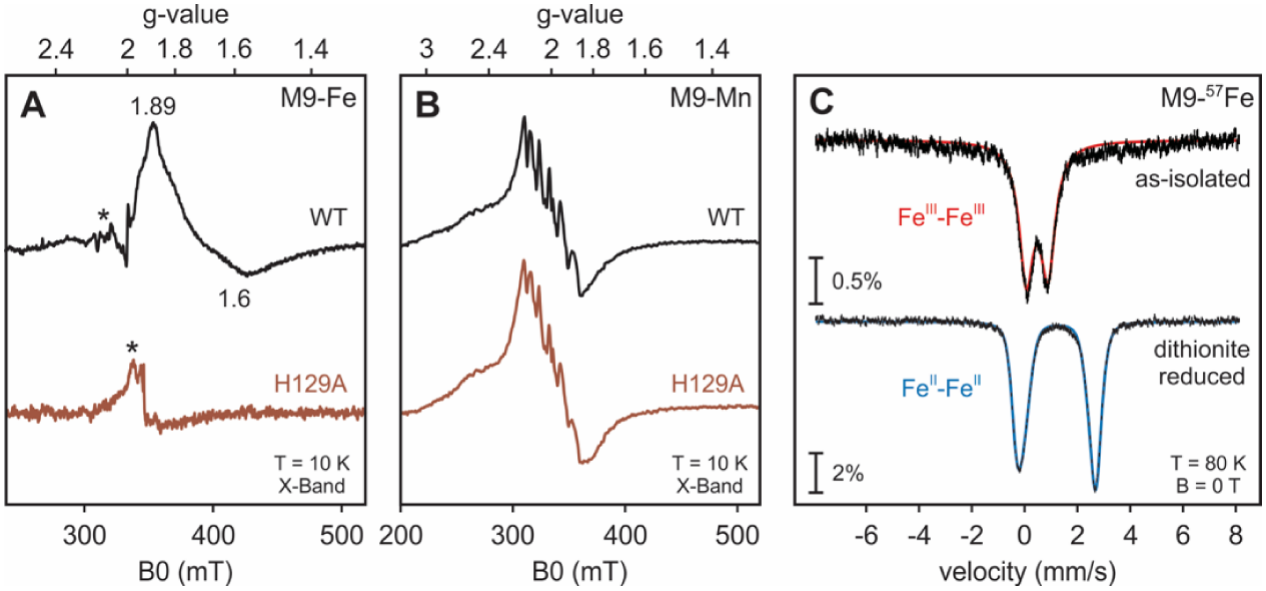
EPR and Mössbauer characterization of *Ri* SAMHD1. (A) CW X-Band EPR spectra of the ascorbate-reduced M9-Fe WT (black) and H129A (brown) *Ri* SAMHD1. Paramagnetic impurities are denoted by asterisks. (B) CW X-Band EPR spectra of the M9-Mn WT (black) and H129A (brown) *Ri* SAMHD1. Experimental conditions: temperature = 10 K, microwave frequency = 9.36 GHz, microwave power = 2 mW, modulation amplitude = 1 mT. (C) ^57^Fe Mössbauer spectra of the as-isolated (top) and dithionite-reduced (bottom) M9-Fe WT *Ri* SAMHD1 recorded at *T* = 80 K and in the absence of an external magnetic field. Experimental spectra are depicted with black solid bars. The red solid line corresponds to the fit of the Fe^III^–Fe^III^ sites, and the blue solid line corresponds to the fit of the Fe^II^–Fe^II^ sites.

Although a second metal ion at the active site was not observed in our crystal structures, *Ri* SAMHD1 retains all five residues necessary for binding a bimetallic cofactor (Figure 1D). To explore this further, *Ri* SAMHD1 was expressed in minimal media supplemented with either Fe or Mn. Elemental analyses showed that the M9-Fe *Ri* SAMHD1 copurifies with 0.97 ± 0.31 molar eq of Fe per monomer, whereas M9-Mn *Ri* SAMHD1 copurifies on average with only 0.18 molar eq of Mn per monomer (Table S4), demonstrating a significantly higher affinity for Fe compared to Mn. EPR spectroscopy shows that the ascorbate-reduced M9-Fe *Ri* SAMHD1 exhibits a broad rhombic signal with principal *g*-values of 1.89 and 1.60 (*g*_av_ < 2) (Figure 2A). Such a signal is reminiscent of an antiferromagnetically (AF) coupled mixed-valent (Fe^II^–Fe^III^, *S* = 1/2) center, confirming assembly of a diiron cofactor.^66−68^ In contrast, the H129A shows no signals suggesting that H129 is a critical residue for the assembly of a dimetal center, similar to what has been reported for the cognate histidine H233 in the human enzyme.^30,34^ On the contrary, Mn enrichment of the samples produces an EPR signal corresponding to a mononuclear rather than a dinuclear Mn^II^ center (Figure 2B).^69,70^ The EPR spectrum along with the extent of Mn incorporation, are essentially identical between WT and H129A M9-Mn *Ri* SAMHD1, indicating that the formation of a dimanganese center is unfavorable, and the copurifying Mn must occupy site 1.^30,34^ These results are consistent with our structural data and may suggest an Fe-centric metal binding cooperativity, in which the affinity for the second metal ion depends on whether metal site 1 is occupied by Fe or Mn.

To further evaluate the extent of diiron site assembly and its redox dependence in the as-purified and dithionite-reduced *Ri* SAMHD1, we employed Mössbauer spectroscopy on samples isolated from minimal media supplemented with ^57^Fe. The spectrum of the aerobically isolated enzyme exhibits a quadrupole doublet that accounts for >85% of the Fe in the sample, and parameters (δ = 0.48 mm/s, Δ*E*_Q_ = 0.82 mm/s) characteristic of nonheme diferric Fe^III^–Fe^III^ sites (Figure 2C).^66−68,71^ Reduction with sodium dithionite results in an upshift in the resonance energies and yields a spectrum that can be best fitted with two quadrupole doublets, with an average isomer shift δ = 1.26 mm/s and quadrupole splitting Δ*E*_Q_ = 2.80 mm/s (Figures 2C, S7). These parameters are characteristic of high-spin Fe^II^ ions (*S* = 2) with N/O coordination, demonstrating homogeneous enrichment of the protein in the Fe^II^–Fe^II^ form.

### Metal-Ion Activated dNTP Hydrolysis by the Tetrameric *Ri* SAMHD1

*Ri* SAMHD1 purifies as a mixture of monomeric and tetrameric forms (Figure S8B, C and D). The presence of a tetrameric form is not contingent upon the addition of exogenous nucleotides or the composition of the expression medium, although its yield varies among different preparations. SEC analysis of concentrated samples of the tetrameric fraction shows little conversion (17%) to the monomer, while the monomeric form does not convert back to the tetramer (Figure S8E). These observations are consistent with the two states being in an apparent equilibrium, likely depending on the binding of a small molecule that is diluted during the chromatographic step. LC/MS analysis of the denatured monomer and tetramer forms confirmed that the tetrameric form copurifies with diphosphate and triphosphate nucleotides (Figure S9A), which promote the observed oligomerization.

**Figure 3:**
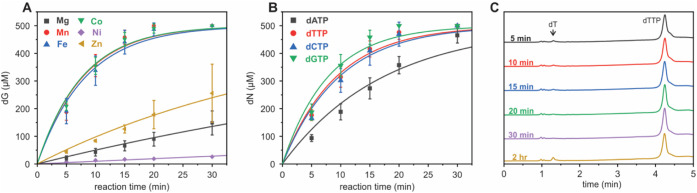
Metal and substrate selectivity of the M9-Fe WT *Ri* SAMHD1 tetramer.(A) dGTP hydrolysis after addition of 1 mM Mg^2+^ (black), Mn^2+^ (red), Co^2+^ (green), Ni^2+^ (purple), Zn^2+^ (gold), or 0.5 mM Fe^2+^ (blue). (B) Time-dependent dNTP hydrolysis in the presence of 1 mM Mn^2+^. (C) HPLC chromatograms monitoring the time-dependent dTTP hydrolysis in the absence of any added divalent metals. *X*-axis is the elution time in minute and *y*-axis is the absorbance at 254 nm. All assays were performed under O_2_-free conditions and reduced by dithionite with a final concentration of 0.5 μM *Ri* SAMHD1 and 0.5 mM dNTP.

The substrate and metal specificity of *Ri* SAMHD1 activity were initially assessed using the tetrameric enzyme isolated from M9-Fe media. The tetramer harbors an intact diiron site and adopts the oligomeric form that is requisite for hydrolysis by all mammalian and eukaryotic SAMHD1s characterized to date.^29,37,62,63^ The activity assays were carried out with dGTP, which serves both as an activator and a substrate for all other previously characterized SAMHD1s.^29,37,62,63^ As expected, dGTP hydrolysis is dependent on the redox state of the diiron cofactor and is maximal after reduction with dithionite that affords the Fe^II^–Fe^II^ form of the cofactor (Figure 2C, S10). All activity assays with the M9-Fe proteins were thus performed under O_2_-free conditions and after reduction with dithionite to ensure homogeneous enrichment in the reduced cofactor. Among the divalent metal ions examined, Fe, Mn, and Co were the most effective activators (Figure 3A, Table 1) irrespective of the nucleotide substrate (Figure S11, Table 1). Substrate specificity of *Ri* SAMHD1 was examined using Mn as an activator, as it provides the same extent of hydrolysis as Fe but is not prone to inadvertent oxidation (Figure S10). The tetrameric form hydrolyzed all four canonical dNTPs, albeit with a slightly different nucleotide preference dGTP > dTTP > dCTP > dATP (Figure 3B, Table 1) compared to that of *Hs* SAMHD1 (dTTP > dATP > dGTP > dCTP).^27,35,72^ Activity assays were also carried out with the M9-Mn *Ri* SAMHD1 and yielded identical results. In addition, activity is premised on an intact bimetallic site, as the H129A variant is hardly active (Figure S12). Therefore, although a dimanganese site was not observed in our EPR and crystallographic investigations, it must form for the enzyme to be active.

**Table 1:** Apparent Rate Constants for dNTP Hydrolysis by WT and Variant *Ri* SAMHD1

Enzyme form	dNTP	Activator	*k*_cat_ (s^–1^)
WT^tet^	dATP	Mn	0.96 ± 0.02
		Fe	0.82 ± 0.01
	dCTP	Mn	1.71 ± 0.03
		Fe	1.36 ± 0.01
	dTTP	-	not detectable
		Mn	1.80 ± 0.14
		Fe	2.40 ± 0.10
		Mg	0.19 ± 0.00
		Co	4.52 ± 0.20
		Ni	0.04 ± 0.00
		Zn	0.44 ± 0.01
	dGTP	Mn	2.12 ± 0.22
		Fe	2.00 ± 0.16
		Mg	0.18 ± 0.01
		Co	2.15 ± 0.15
		Ni	0.03 ± 0.00
		Zn	0.36 ± 0.02
WT^mon^	dATP	Mn	0.49 ± 0.01
		Fe	0.37 ± 0.00
	dCTP	Mn	0.36 ± 0.00
		Fe	0.34 ± 0.00
	dTTP	Mn	0.64 ± 0.01
		Fe	0.80 ± 0.02
	dGTP	Mn	1.02 ± 0.06
		Fe	1.00 ± 0.08
D34A^mon^	dATP	Mn	0.45 ± 0.02
		Fe	0.35 ± 0.01
	dCTP	Mn	0.27 ± 0.01
		Fe	0.29 ± 0.01
	dTTP	Mn	0.42 ± 0.00
		Fe	0.96 ± 0.02
	dGTP	Mn	1.44 ± 0.03
		Fe	0.72 ± 0.10

Curiously, dTTP hydrolysis required the addition of exogenous metals (Figure 3C), which is surprising given that the enzyme (i) has an intact bimetallic cofactor and, (ii) is already in the tetrameric form necessary for activity.^29,37,62,63^ This unexpected need for exogenous metals suggests the presence of an additional metal-centered regulatory site, separate from the two known allosteric sites at the tetramerization interface.^27,34,73^ It is possible that a similar requirement exists in the human enzyme, but it may have been obscured due to the coupling of allostery and catalysis.^27,28,30^ This additional metal site could not be identified in our crystallographic experiments but may correspond to the third Mg ion observed in the structures of the human enzyme, where the extra Mg coordinates the phosphate substrate oxygens (Figure 1D).^35^

### dNTP Hydrolysis by *Ri* SAMHD1 Is Not Allosterically Regulated

**Figure 4:**
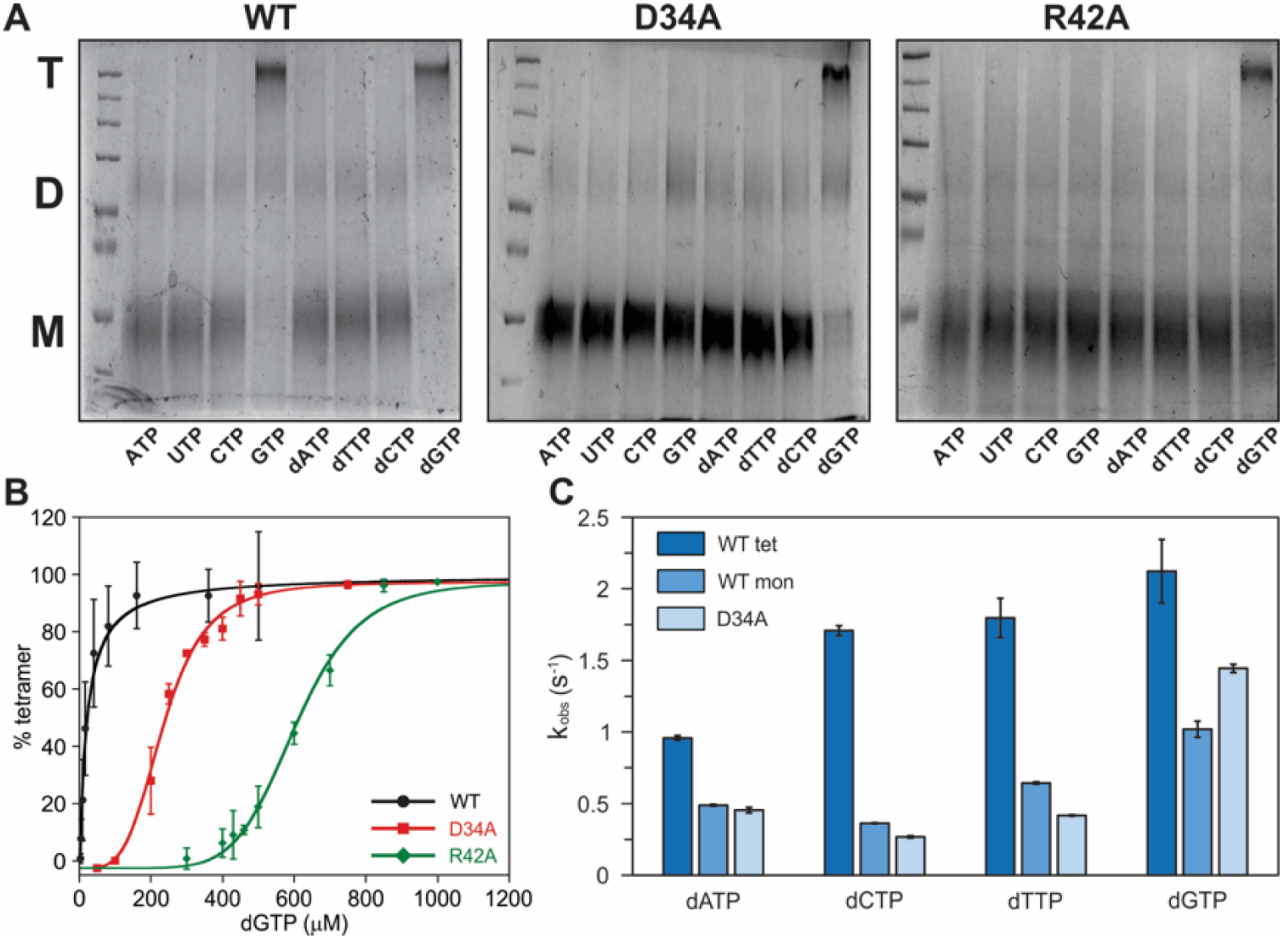
Regulation of allostery and activity in *Ri* SAMHD1. (A) Chemical cross-linking gels of the WT monomer (left) and the AL1 variants D34A (middle) and R42A (right) of *Ri* SAMHD1. 2 μM protein was incubated with the desired (d)NTP (500 μM) and Mn (1 mM) followed by incubation with 20 mM glutaraldehyde. The positions of monomer (M), dimer (D), and tetramer (T) are indicated. (B) Tetramerization of the monomer WT *Ri* SAMHD1 (black) and the AL1 variants D34A (red) and R42A (green) as a function of dGTP concentration. 2 μM protein was incubated with dGTP (0.002 to 1 mM) and Mn (1 mM) followed by incubation with 20 mM glutaraldehyde. The percent formation of tetramer was quantified by estimating the relative intensity of the gel bands using ImageJ. (C) dNTP hydrolysis by the WT monomer and tetramer *Ri* SAMHD1 and the D34A variant in the presence of 1 mM Mn. All assays were performed under O_2_-free conditions. Experimental conditions: [*Ri* SAMHD1] = 0.5 μM, [dithionite] = 5 mM, [dNTP] = 0.5 mM.

*Ri* SAMHD1 shares an identical AL1 (DXXQXXXR) and a highly similar AL2 with *Hs* SAMHD1 (Figure 1B, D), suggesting that both proteins are allosterically regulated in a similar manner. To test this hypothesis, we carried out chemical cross-linking experiments in which the monomeric *Ri* SAMHD1 was incubated with different nucleotides and Mn. *Ri* SAMHD1 formed tetramers only in the presence of GTP or dGTP, but not with any other nucleotides (Figure 4A), indicating a similar requirement to that of *Hs* SAMHD1 for a guanosine-containing effector.^36,74^ One distinct difference, however, is that in *Ri* SAMHD1, addition of GTP does not lead to the accumulation of a detectable dimeric intermediate; instead, the enzyme directly transitions to the tetrameric form. Furthermore, oligomerization does not exhibit specificity for the type of allosteric metal ion, as *Ri* SAMHD1 tetramerizes with all divalent metal ions examined (Figure S13).

The role of AL1 in mediating this allosteric transition in *Ri* SAMHD1 was interrogated using alanine variants of two out of the three residues of AL1, D34, and R42, both of which interact with the GTP guanine base (Figure 1B, D).^30^ The D32A and R42A *Ri* SAMHD1 variants purify as monomers, and not oligomeric mixtures, highlighting the role of these residues in oligomerization. Interestingly, however, the D34A and R42A variants can tetramerize with dGTP (but not GTP), but at much higher nucleotide concentrations than the WT protein (Figure 4A). In the WT, dGTP-induced tetramerization showed no cooperativity and exhibited a *K*_tet_ of 21 ± 3 μM (Figure 4B), which was essentially the same as that estimated for GTP (*K*_tet_ = 31 ± 2 μM) (Figure S14). In contrast, the dGTP-induced tetramerization of the D34A and R42A *Ri* SAMHD1 variants showed cooperativity, with apparent *K*_tet_ values of 235 ± 5 and 605 ± 9 μM, respectively (Figure 4B). These results demonstrate that AL1 mutations impair enzyme tetramerization and that GTP (but not dGTP) binding requires an intact AL1 site. As expected, because these residue substitutions are distant from the hydrolytic site, they do not impact assembly of the bimetallic cofactor (Figure S15).

Our results show that tetramerization of *Ri* SAMHD1 is driven by a G-effector, similar to *Hs* SAMHD1, but do not determine whether oligomerization is required for activity. To explore this, we employed the monomeric form of *Ri* SAMHD1 and tested its ability to hydrolyze dNTPs without a G-effector. Curiously, the enzyme shows significant activity with dATP, dCTP, and dTTP in the absence of GTP, which contrasts with the human enzyme that is completely inactive under the same conditions.^31,37,40,74^ The monomeric D34A variant is as active as the WT protein, further confirming that alterations in AL1 do not impact hydrolysis. Activity of the monomer is appreciable and comparable to that reported for the tetrameric forms of mammalian and eukaryotic SAMHD1s.^35^ Addition of GTP increases the apparent hydrolysis rates by up to 2.4-fold, indicating that tetramerization activates the enzyme, though only to a modest degree. dGTP is an exception as it acts both as an effector and a substrate, yielding apparent catalytic rates of ca. 1 s^–1^ (Figure 4A), which is approximately half of that observed for the as-purified tetramer (Figure 4C, Table 1). LC/MS analysis of the purified tetramer, chemical cross-linking, and activity assays, uncover the copurification of GTP and GDP as the molecular basis for the enhanced activity (Figure S9B, C). It is important to note that hydrolysis rates in our studies were independent of the divalent metal ion used as an activator (i.e., Mn or Fe) (Figure S16, Table 1).

### An Opposing Dichotomy in Activity Regulation of *Ri* SAMHD1 by GTP and dGTP

**Figure 5:**
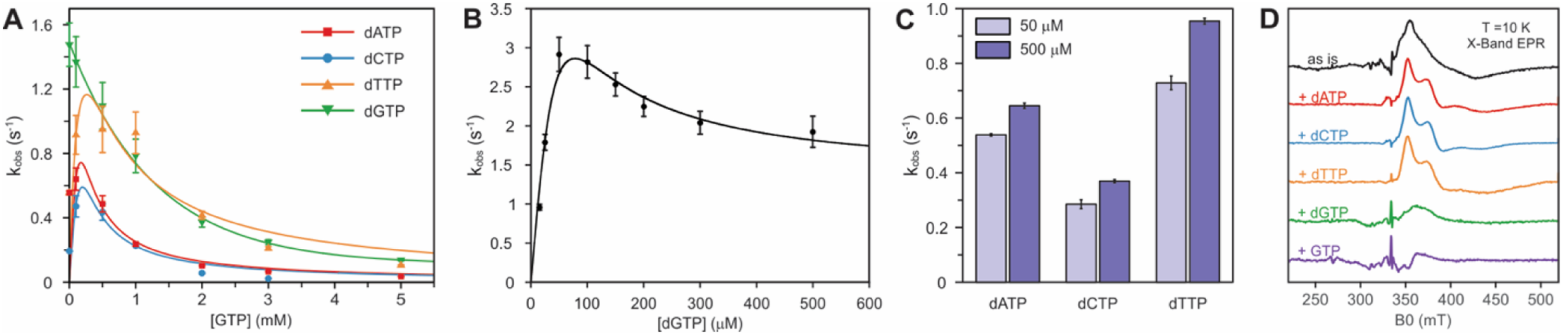
GTP and dGTP regulate *Ri* SAMHD1 activity. (A) Observed dNTP hydrolysis rate of M9-Fe WT *Ri* SAMHD1 monomer at varying GTP concentrations (0 to 5 mM). All assays were performed under O_2_-free conditions. Experimental conditions: [*Ri* SAMHD1] = 0.5 μM, [dithionite] = 5 mM, [Mn] = 1 mM. (B) Steady-state kinetics of M9-Fe WT *Ri* SAMHD1 tetramer with dGTP. Data are fitted with the Michaelis–Menten equation considering substrate/product inhibition using eq 2. (C) Observed dNTP hydrolysis rate of M9-Fe WT *Ri* SAMHD1 tetramer with 50 μM and 500 μM dNTP. All assays in (B) and (C) were performed under O_2_-free conditions. Experimental conditions: [*Ri* SAMHD1] = 0.25 μM, [dithionite] = 5 mM, [Mn] = 1 mM. (D) EPR spectra monitoring different nucleotides binding to M9-Fe WT *Ri* SAMHD1. All samples were made under O_2_-free conditions and were reduced using ascorbate acid for 10 min. Experimental conditions: [*Ri* SAMHD1] = 0.5 mM, [ascorbate] = 1 mM, [GTP/dNTP] = 2.5 mM.

To further investigate the efficiency of GTP in enhancing *Ri* SAMHD1 activity, we examined hydrolysis by the monomeric form as a function of varying effector concentrations and dNTPs. With all nonguanine-containing dNTPs, GTP initially exerts an activating effect at low concentrations (<0.2 mM) but transitions to an antagonistic effect at higher concentrations (>0.2 mM) (Figure 5A). For GTP concentrations up to 1.5 mM, activity reaches that of the monomeric enzyme, while at higher GTP concentrations, complete inhibition is observed (eq 3, Table S4). With dGTP, no activating effect is observed; instead, activity consistently decreased at higher GTP levels (Figure 5A), presumably due to the additive effects of the two G-based nucleotides. These results demonstrate that GTP functions as both an effector and an inhibitor, a behavior that markedly differs from that of *Hs* SAMHD1, for which GTP exerts only a stimulatory effect, even at concentrations up to 5 mM.^39^ These observations highlight a divergence in the regulation of *Ri* SAMHD1 activity and prompt further investigation into the chemical nature of the inhibition, whether allosteric or competitive.

To further evaluate the extent of dGTP inhibition, experiments were carried out with the isolated tetramer form. While dGTP concentrations up to approximately 100 μM resulted in enhanced apparent hydrolysis rates, higher dGTP concentrations led to reduced activity (Figure 5B). The apparent Michaelis constant (*K*_M_) was estimated to be 11 ± 3 μM and the apparent inhibition constant (*K*_I_) 301 ± 24 μM. The inhibitory effect was specific to dGTP and was not observed with any of the three other nucleotides (dATP, dCTP, and dTTP), which instead, enhanced hydrolysis rates at higher substrate concentrations (Figure 5C).

To gain further insight into the molecular basis of this apparent inhibition by (d)GTP, we monitored whether addition of dNTPs exerts changes on the line shape and g-values of the mixed-valent cofactor EPR signal (Figure 5D). Addition of nonguanine-containing dNTPs (dATP, dCTP, and dTTP) increased the signal rhombicity and produced g-value shifts, indicative of substrate binding at the active site (Figure 5D). In contrast, addition of GTP and dGTP led to a decrease or complete disappearance of the mixed-valent Fe^II^–Fe^III^ signal. The observation that only addition of (d)GTP causes a pronounced loss of the EPR signal, and not any of the other nucleotides, suggests that the inhibition caused by G-containing effectors or substrates likely occurs at the allosteric level, inducing conformational changes that inactivate the protein rather than (d)GTP competitively binding at the active site.

### Phylogenetic Distribution of SAMHD1

**Figure 6:**
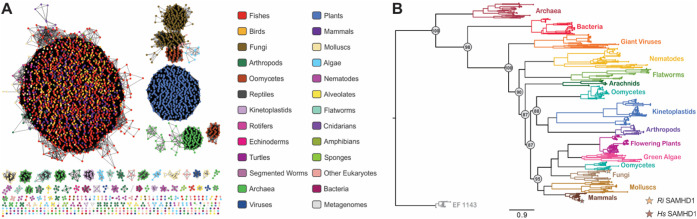
Occurrence and phylogenetic distribution of SAMHD1. (A) SSN of SAMHD1. The SSN contains 4,466 unique sequences, with nodes colored based on taxonomic class. (B) The maximum likelihood phylogenetic tree of SAMHD1 was generated with the IQ-tree software using the LG+R10 model, with the EF1143 dNTPase used as the outgroup. The scale bar represents the number of substitutions per site. The bootstrap values are shown for the major branches. The positions of *Ri* SAMHD1 and *Hs* SAMHD1 in the tree are denoted with stars.

To explore the diversity and evolutionary relationships within the SAMHD1 superfamily, we generated a sequence similarity network (SSN) using *Hs* SAMHD1 as the input in a sequence BLAST (Figure 6A).^53,54^ The resulting SSN contains 4,466 unique sequences and 3,428 nodes, with each node representing proteins that share at least 95% sequence identity. The sequences are segregated into 311 clusters, with 137 clusters containing more than one sequence. The majority of the sequences originate from eukaryotic organisms (89.2%), while SAMHD1 is also present in archaea (7.8%), giant viruses (0.96%), and bacteria (1.3%). The two largest clusters of the SSN account for 63% of the total sequences, corresponding to vertebrates (fish, birds, mammals, etc.) and plants. All other identified SAMHD1 sequences differ from the mammalian ones in at least three characteristic regulatory components: the absence of the N-terminal SAM domain, a lack or minimal conservation of AL2, and the absence of the three cysteines that regulate redox activity.

We carried out a phylogenetic analysis to elucidate the evolutionary relationships between SAMHD1 proteins and the diversification of their regulatory sites.^73^ The maximum-likelihood phylogenetic tree, which included 451 unique SAMHD1 sequences, was rooted using the distantly homologous HD-domain protein EF1143 as an outgroup (Figure 6B).^57^ EF1143 is a dNTPase and, similar to *Hs* SAMHD1, is active as a tetramer, unlike other HD-domain dNTPases (often mistakenly referred to as SAMHD1-like) that are active as hexamers.^57,75,76^ The phylogenetic tree reveals that SAMHD1 first emerged in archaea, bacteria, and giant viruses, subsequently appearing in parasites such as nematodes, flatworms, oomycetes, arthropods, and kinetoplastids. Finally, SAMHD1 emerged in more sophisticated organisms, including plants, algae, fungi, mollusks, and mammals.^19−22,24^ Phylogenetically, *Ri* SAMHD1 branches out from the mammalian proteins and clusters with plant SAMHD1 orthologs, which have been suggested to possess dNTPase activity linked to photosynthetic fitness *in vivo*.^19−22^ This segregation suggests that the fungal protein may serve as a functional homologue to the plant proteins, which to date have resisted biochemical characterization.

## Discussion

SAMHD1 is a hydrolase crucial for regulating dNTP homeostasis in humans and other eukaryotic organisms, including animals, plants, and parasites.^19−25,63^ While SAMHD1-related pathways and functions are well characterized in humans, its roles in other species are less well understood. In plants, *samhd1* mutations compromise photosynthetic ability due to defects in chlorophyll biosynthesis and chloroplast ribosome biogenesis. In the human parasite *Tb,* deletion of *samhd1* renders the parasite a pyrimidine auxotroph and confers genome integrity defects. Despite the critical role of SAMHD1 orthologs in organism fitness, their biochemical intractability has hindered their molecular characterization.^19−25^ As a result, the SAMHD1 superfamily remains an unmapped protein space with respect to substrate scope, metal dependence, and allosteric regulation. In this study, we bioinformatically mined a large set of SAMHD1s with an unrecognized functional repertoire. Their phylogenetic occurrence suggests that they capture evolutionary events predating the appearance of the human enzyme and highlight a species divergence in metal utilization and allosteric mechanisms. We selected the SAMHD1 from the mycorrhizal fungus *Ri* as a model system to investigate dNTP hydrolysis in plants and mutualistic soil fungi, and to gain insight into their functional differentiation from the mammalian enzymes.

Our bioinformatic analyses show that *Ri* SAMHD1 lacks the nuclear localization domain, suggesting that it is not imported into the nucleus. Additionally, *Ri* SAMHD1, like all nonmammalian orthologs, lacks the SAM domain, a module commonly found in eukaryotic organisms and primarily involved in cell signaling due to its oligomerization properties.^77^ Although the SAM domain in *Hs* SAMHD1 is not essential for retroviral restriction, oligomerization, or RNA binding,^78^ it promotes tetramer stabilization and dNTP hydrolysis in mice.^29^ Despite the absence of the SAM N-terminal motif, *Ri* SAMHD1 copurifies as a mixture of monomeric and tetrameric forms, indicating that the lack of this domain does not disfavor oligomerization.

In *Ri* SAMHD1, the allosteric sites are similarly arranged to those in *Hs* SAMHD1, with two notable differences: the presence of a Ca^2+^ ion instead of Mg^2+^ or Mn^2+^, and a single nucleotide effector, rather than two. Evidence suggests that this structure, with a single GTP at the tetramer interface, may depict a biologically relevant state. In fact, tetramer formation is promoted by very low GTP concentrations suggesting that under cellular conditions the protein is a constitutive tetramer. In addition, all allosteric residues interacting with GTP are similarly arranged to the cognate ones in tetrameric *Hs* SAMHD1.^28,33,34^ However, GTP alone is unable to tetramerize the human enzyme without an ancillary dNTP, even at concentrations exceeding the reported cellular amounts,^79^ marking a distinct difference with the fungal enzyme. Although our current data suggest that our crystal structure is a biologically relevant form, we cannot exclude the possibility that this single-nucleotide state represents a partially inhibited form or a crystallization artifact.

Excluding the SAM domain, *Ri* SAMHD1 shares high sequence similarity both with *Hs* SAMHD1 as well as the plant SAMHD1 proteins. *Ri* SAMHD1 conserves all five active site residues involved in the coordination of a bimetallic center invoking a common two-metal-based mechanism for the stabilization of a hydroxyl nucleophile to initiate cleavage of the organophosphate bond.^34^ Indeed, Fe-enriched *Ri* SAMHD1 assembles a diiron cofactor, which can exist in three oxidation states: the Fe^III^–Fe^III^, the partially reduced Fe^II^–Fe^III^, and the fully reduced Fe^II^Fe^II^ forms. This is the first demonstration of a diiron site in SAMHD1 proteins, as the human enzyme contains Fe in only one of the sites.^35^ The Fe^III^–Fe^III^ form shows minimal activity and only when the enzyme is treated with sodium dithionite is fully activated, demonstrating that the diferrous cofactor represents the catalytically active form. Curiously, Mn-enriched *Ri* SAMHD1 does not purify with a dimanganese cofactor, as demonstrated by both EPR and crystallographic experiments. Despite the inability to spectroscopically detect a dimanganese site, dNTP hydrolysis is premised on a Mn^II^–Mn^II^ center, as evidenced by the impaired activity of the H129A Mn-enriched *Ri* SAMHD1 variant. These results collectively demonstrate that (i) dNTP hydrolysis by *Ri* SAMHD1 proceeds via a two-metal-based mechanism, and (ii) Mn binding at site 1 decreases the apparent affinity for a metal ion at site 2, unlike the case of Fe, suggesting an Fe-dependent cooperativity for active-site assembly.

In the absence of exogenously added nucleotides, the as-purified *Ri* SAMHD1 exists as a mixture of monomeric and tetrameric forms. The tetramer is stabilized by copurifying (d)GTP nucleotides and likely accumulates due to their higher affinity for promoting tetramerization, compared to the human enzyme.^39^ Monomeric *Ri* SAMHD1 requires at least one G-effector for tetramerization, which is not limited to triphosphates, but also includes mono- and diphosphate G-based nucleotides, similar to what has been observed for the human enzyme.^74^ Curiously, activity is weakly dependent on oligomerization and the monomer is only 2- to 5-fold less active compared to the tetramer form. Considering that monomeric *Ri* SAMHD1 hydrolyzes dNTPs at a rate comparable to that of the allosterically activated human SAMHD1,^1,27,35^ the modest rate enhancement by tetramerization is unlikely to play a significant regulatory role in *Ri* SAMHD1. However, if this activity stimulation is biologically relevant, GTP and GDP are the most likely activators for *Ri* SAMHD1, given their cellular concentrations.^79^ Notably, the ability of *Ri* SAMHD1 to hydrolyze dNTPs in its monomeric form reveals that its active site does not undergo any substantial structural rearrangements that would significantly impact activity, unlike the human enzyme, in which tetramerization induces a more compact active site conformation, facilitating hydrolysis.^36,40^

While the purified tetrameric *Ri* SAMHD1 has an intact bimetallic site and satisfies all known requirements for catalysis, it is not competent for dNTP hydrolysis without exogenous addition of metal ions. Interestingly, activity still shows a strict dependence on exogenous metal ions, suggesting the presence of an additional, yet unidentified, metal requirement for catalysis beyond active site assembly and oligomerization. This activation requirement is likely also operative in the human enzyme, although it may be skewed by the strong coupling between allostery and catalysis.^37,74^ Given the absence of any other predicted metal-binding domains and the observation that a Mg ion – distinct from the bimetallic catalytic center – coordinates the β- and γ-phosphates of the nucleotide substrate in the available crystal structures of the human enzyme,^34^ it is tempting to propose that the ancillary metal requirement for *Ri* SAMHD1 activation involves a third metal ion located near the active site, which solely interacts with the substrate phosphates. Furthermore, only transition metal ions were effective in stimulating dNTP hydrolysis in *Ri* SAMHD1, while Mg was a poor activator. These findings suggest that allosteric activation by Mg may be a feature acquired later in the more mechanistically advanced human enzyme. The facile formation of a diiron site and the effectiveness of Fe as an activator further underscore the critical role of this redox-metal ion in the *Ri* SAMHD1 activity. The dependence on Fe or Mn for SAMHD1 activity is not surprising, as *Ri* possesses specialized machinery for Fe acquisition to enhance plant iron uptake, while also contributing to plant health under Mn-toxic conditions.^80,81^

Although allostery is the primary regulatory mechanism modulating *Hs* SAMHD1 activity, other factors such as redox regulation and post-translational modifications, also play a role.^42,44^ In this regard, *Ri* SAMHD1 lacks the three cysteines proposed to function as an intramolecular disulfide redox-switch, as well as C-terminal residues that undergo post-translational modifications.^42,44^ This suggests that the regulation of activity in the fungal ortholog is either less stringent or modulated through alternative molecular mechanisms. Indeed, we observed that GTP acts both as an allosteric effector and as an inhibitor at concentrations exceeding 0.2 mM. While the precise molecular mechanism of inhibition could not be definitively resolved in our activity assays or spectroscopic experiments, our data suggest that GTP functions as an allosteric inhibitor. This mode of inhibition is not operative in the human enzyme, in which GTP can stimulate dNTP hydrolysis up to concentrations of 5 mM.^39^ The cellular (d)NTP concentrations in *Ri* are not yet known, but the typical GTP levels in eukaryotic cells range from 0.25 to 0.7 mM,^79^ which would make GTP a likely inhibitor under certain conditions. It is intriguing to consider that a potential regulatory mechanism for dNTP hydrolysis in *Ri* could involve downregulation of activity, as the modest rate enhancement observed at lower GTP concentrations suggests a relatively loose activation control. Further experiments are needed to determine whether this GTP-dependent fine-tuning of activity plays a significant role in *Ri* nucleotide metabolism.

Although *Ri* SAMHD1 hydrolyzes all canonical deoxynucleotides, it exhibits a preference for dGTP, which could lead to the overproduction of dG and inorganic triphosphate. Interestingly, dG derived from endophytic fungal metabolites has been shown to trigger an immune response in plants, although its precise origin remains unclear.^47^ It can thus be speculated that the triphosphohydrolase activity of *Ri* SAMHD1 serves a dual role: on one hand, contributing to nucleotide homeostasis within the fungus, and on the other, influencing plant immunity through the production of dG and phosphate. As *Ri* is a common symbiont of plants, a deeper understanding of these regulatory dynamics could provide valuable insights into the interplay between fungal metabolites and plant immune responses, shedding light on the complex mutualistic relationship between endophytic fungi and their host plants.

## Supporting Information Available

Additional experimental details, materials, and methods; Figures S1–S3 show structural comparisons of *Ri* SAMHD1 and *Hs* SAMHD1, overall topology, sequence alignments, and RMSD-based mapping; Figures S4–S6 highlight active and allosteric site coordination, metal ion binding, and oligomerization interfaces; Figure S7 shows Mössbauer spectra; Figure S8 includes SDS-PAGE and SEC analyses; Figures S9–S11 show biochemical assays examining copurifying molecules, redox effects on dNTP hydrolysis, and metal-dependent catalysis; Figures S12–S16 explore active site mutations, nucleotide-driven tetramerization, and EPR spectra; the supplemental tables provide primer sequences, crystallographic data, sequence alignments, elemental composition, and kinetic parameters (PDF)
