# Supplementary material for: Activation and Allostery in a Fungal SAMHD1 Hydrolase: An Evolutionary Blueprint for dNTP Catabolism
Source: JACS Au. 2025 Apr 17;5(4):1862–74. doi: 10.1021/jacsau.5c00090 (PMC12042053; doi:10.1021/jacsau.5c00090)
Supplement: Supplementary file 1 — au5c00090_si_001.pdf [file au5c00090_si_001.pdf]

Activation and allostery in a fungal SAMHD1 hydrolase; an evolutionary blueprint for dNTP catabolism

Supporting Information

Luying Pan[1], Jake Lachowicz [2], Isaac Paddy [1], Yutong Xu[1], Qianyi Yang[1], Cynthia Zizola [2], Amy Milne [1], Tyler L. Grove[2,], Maria-Eirini Pandelia[1,*]*

[1]Department of Biochemistry, Brandeis University, Waltham, Massachusetts 02453, United States

[2] Department of Biochemistry, Albert Einstein College of Medicine, Bronx, NY 10461, USA

*Corresponding authors: Maria-Eirini Pandelia, Tyler L. Grove

Email: mepandelia@brandeis.edu

tyler.grove@einsteinmed.edu

KEYWORDS: HD-domain, iron, manganese, allostery, *Rhizophagus irregularis*

Supplemental Materials and Methods

Protein expression and purification

All plasmids were transformed into T7 express *Escherichia coli* competent cells (New England Biolabs, MA) and selected for kanamycin resistance. To direct specific metal incorporation, transformed cells were grown in minimal (M9) media in the presence of 0.2% (v/v) glucose, 0.1 mM $CaCl_2$, 200 mM $MgSO_4$, and different transition metal ion salts: Fe^{2+} (125 µM $(NH_4)_2Fe(SO_4)_2$), or Mn^{2+} (100 µM $MnCl_2$) until an OD_{600} of 0.6-0.8 was reached. The cultures were cold shocked for 1 hour at 4 °C, protein expression was induced by addition of 0.5 mM Isopropyl β-d-1-thiogalactopyranoside (IPTG) and different transition metal ion salts: Fe^{2+} (125 µM $(NH_4)_2Fe(SO_4)_2$), or Mn^{2+} (100 µM $MnCl_2$). Cell cultures were incubated at 18 °C with shaking (200 rpm) for 16-20 hours. Cells were harvested by centrifugation at 1,600 x g and 4 °C for 20 min, flash frozen in liquid nitrogen, and stored at -80 °C. Cell pellets were resuspended in lysis buffer (50 mM 2-morpholinoethanesulfonic acid hydrate (MES), 50 mM L-arginine hydrochloride (L-arginine HCl), 300 mM sodium chloride (NaCl), 10 mM imidazole, 0.5 mM tris(2-carboxyethyl) phosphine (TCEP), pH 7.0) and lysed using a Microfluidizer LM20 (Microfluidics) with the addition of 10 mg/L lysozyme, 10 mg/L DNase, and 0.45 µg/L phenylmethylsulfonyl fluoride (PMSF) prior to cell disruption. Lysed cells were centrifuged at 40,000 x g for 30 min. The clarified lysate was loaded onto a Ni^{2+}-NTA immobilized affinity chromatography column (~10 mL resin per 100 mL lysate) equilibrated with the lysis buffer. The column was first washed with lysis buffer and then with wash buffer (50 mM MES, 50 mM L-arginine HCl, 300 mM NaCl, 30 mM imidazole, 0.5 mM TCEP, pH 7.0). The protein was eluted with elution buffer (50 mM MES, 50 mM L-arginine HCl, 150 mM NaCl, 250 mM imidazole, 0.5 mM TCEP, pH 7.0) and mixed with an equal volume of storage buffer (50 mM MES, 50 mM L-arginine HCl, 150 mM NaCl, 10 % glycerol, pH 6.5) to lower the imidazole and minimize protein precipitation. The elution was concentrated at 3,900 rpm using a 50 K Amicon Centrifugal Filter Unit (Millipore Sigma, MA). The concentrated protein was loaded onto a size exclusion HiLoad 16/600 Superdex 200 pg column (Cytiva, MA), which was equilibrated with storage buffer. Fractions containing the pure protein of interest were pooled and further concentrated. Protein purity was estimated by SDS-PAGE with Coomassie staining, and protein concentration was determined using a molar absorption coefficient of 51,230 M^{-1} cm^{-1} at 280 nm (https://web.expasy.org/protparam/) via Ultraviolet-visible (UV-Vis) spectrophotometer (Agilent, CA). The protein was flash-frozen in liquid nitrogen and stored at -80 °C prior to further use.

Size Exclusion Chromatography

A 100 µL sample of 30 µM *Ri* SAMHD1 was injected into a Superdex 200 Increase 10/300 GL column (Cytiva). The column was equilibrated in storage buffer (50 mM MES hydrate, 50 mM L-arginine HCl, 150 mM NaCl, 10 % glycerol, pH 6.5). The fractions corresponding to the monomer or tetramer were combined, concentrated at 3,900 rpm using a 50 K Amicon Centrifugal Filter Unit (MilliporeSigma, MA), and re-injected again to examine the stability of the oligomeric state of *Ri* SAMHD1.

Activity assays

Time-dependent reactions to examine the exogenous metal selectivity of *Ri* SAMHD1 were carried out with 0.5 µM of the tetrameric M9-Fe WT *Ri* SAMHD1 and a series of transition metal ion salts: Fe^{2+} (0.5 mM $(NH_4)_2Fe(SO_4)_2$), Mn^{2+} (1 mM $MnCl_2$), Mg^{2+} (1 mM $MgCl_2$), Ni^{2+} (1 mM $NiCl_2$), Co^{2+} (1 mM $CoCl_2$), and Zn^{2+}

(1 mM $ZnSO_4$). Reactions were initiated by the addition of 0.5 mM dTTP or dGTP and quenched after 5, 10, 15, 20, and 30 min. Time-dependent reactions to examine the activity of tetrameric *Ri* SAMHD1 in the absence of any divalent metals were carried out with 0.5 µM of the tetrameric M9-Fe WT *Ri* SAMHD1. Reactions were initiated by the addition of 0.5 mM dTTP and quenched after 5, 10, 15, 20, 30, and 120 min. Time-dependent reactions to examine the substrate specificity of *Ri* SAMHD1 were carried out with 0.5 µM of the monomeric WT, tetrameric WT, and D34A *Ri* SAMHD1 expressed in M9-Fe media, in the presence of 0.5 mM $(NH_4)_2Fe(SO_4)_2$ or 1 mM $MnCl_2$. Reactions were initiated by the addition of 0.5 mM dNTP (dATP, dTTP, dCTP, or dGTP) and quenched after 5, 10, 15, 20, and 30 min. Time-dependent reactions to examine *Ri* SAMHD1 dNTP hydrolysis under different GTP concentrations were carried out with 0.5 µM monomeric WT M9-Fe *Ri* SAMHD1, 1 mM $MnCl_2$, and 0-5 mM GTP. Reactions were initiated by the addition of 0.5 mM dNTP (dATP, dTTP, dCTP, or dGTP) and quenched after 5, 10, 15, and 20 min. Estimation of the steady-state parameters was carried out in multiple turnover assays using 0.15-1 mM dGTP as the substrate and including 0.25 µM of the tetrameric WT M9-Fe *Ri* SAMHD1 and 1 mM $MnCl_2$ with the reaction time 5, 10, 15, 20, and 30 min. All reactions were carried out under O_2-free conditions in an anaerobic glovebox in the storage buffer. Reactions were quenched at the described time points by addition of 1 eq volume of 100 mM potassium hydroxide (KOH). The initial rates were fitted by linear regression to a first-order reaction.

$$[P] = [S]_0(1 - e^{-\frac{v}{E_0}t}) \qquad \text{(Equation 1)}$$

The initial rates in the dGTP titrations were fitted with the Michaelis-Menten equation considering partial substrate inhibition:[1]

$$\frac{v}{E_0} = \frac{k_{cat}\cdot[S]}{K_1+[S]+\frac{[S]^2}{K_2}} + \frac{k'_{cat}\cdot[S]}{\frac{K_1\cdot K_2}{[S]}+K_2+[S]} \qquad \text{(Equation 2)}$$

The initial rates in the GTP titrations were fitted with the Michaelis-Menten equation considering substrate inhibition:

$$\frac{v}{E_0} = \frac{k_{cat}\cdot[S]}{K_1+[S]+\frac{[S]^2}{K_2}} \qquad \text{(Equation 3)}$$

The data were fitted using the Origin 2021 and the Kaleidagraph software.

M9-Fe and M9-Mn *Ri* SAMHD1 redox assays

Hydrolysis of dGTP was examined under three different redox conditions in the presence of 1 mM $MnCl_2$: as isolated, reduced (treated with 5 mM dithionite for 15 min), and re-oxidized (reduced protein treated with 10 mM H_2O_2 for 15 min). Reduction with dithionite and activity of the reduced *Ri* SAMHD1 were carried out under O_2-free conditions in the glovebox. Reactions were initiated by the addition of 0.5 mM dGTP and quenched at 20 min by addition of 1 eq volume of 100 mM potassium hydroxide (KOH).

Supplemental Figures

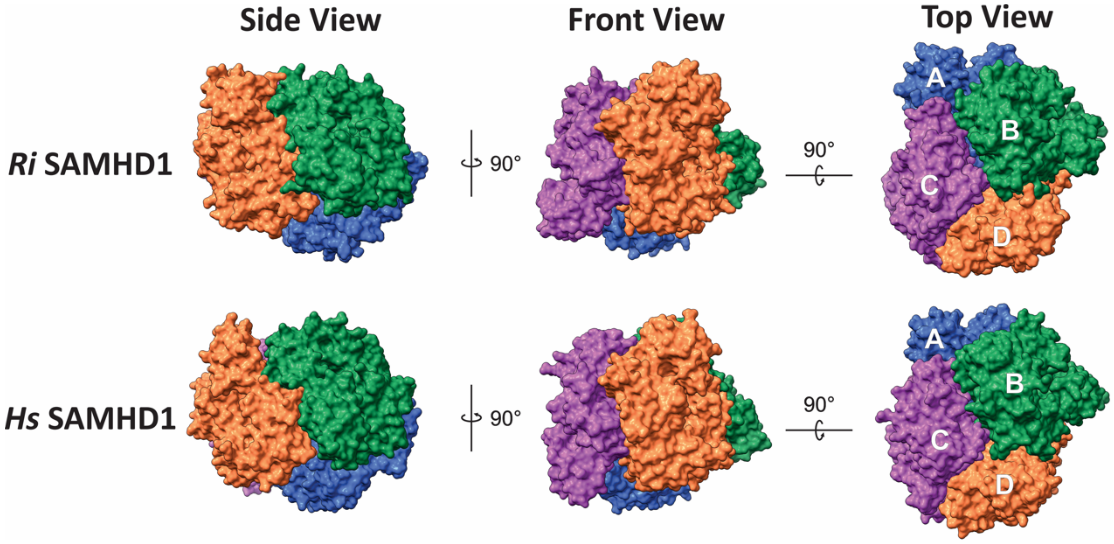

Figure S1.

Surface representations of the tetrameric *Ri* SAMHD1 (PDB: 9MR6) and *Hs* SAMHD1 (PDB ID: 7A5Y) depicted overall structure in three orthodiagraphic views. Subunits A, B, C, and D have been colored blue, green, purple, and orange, respectively.

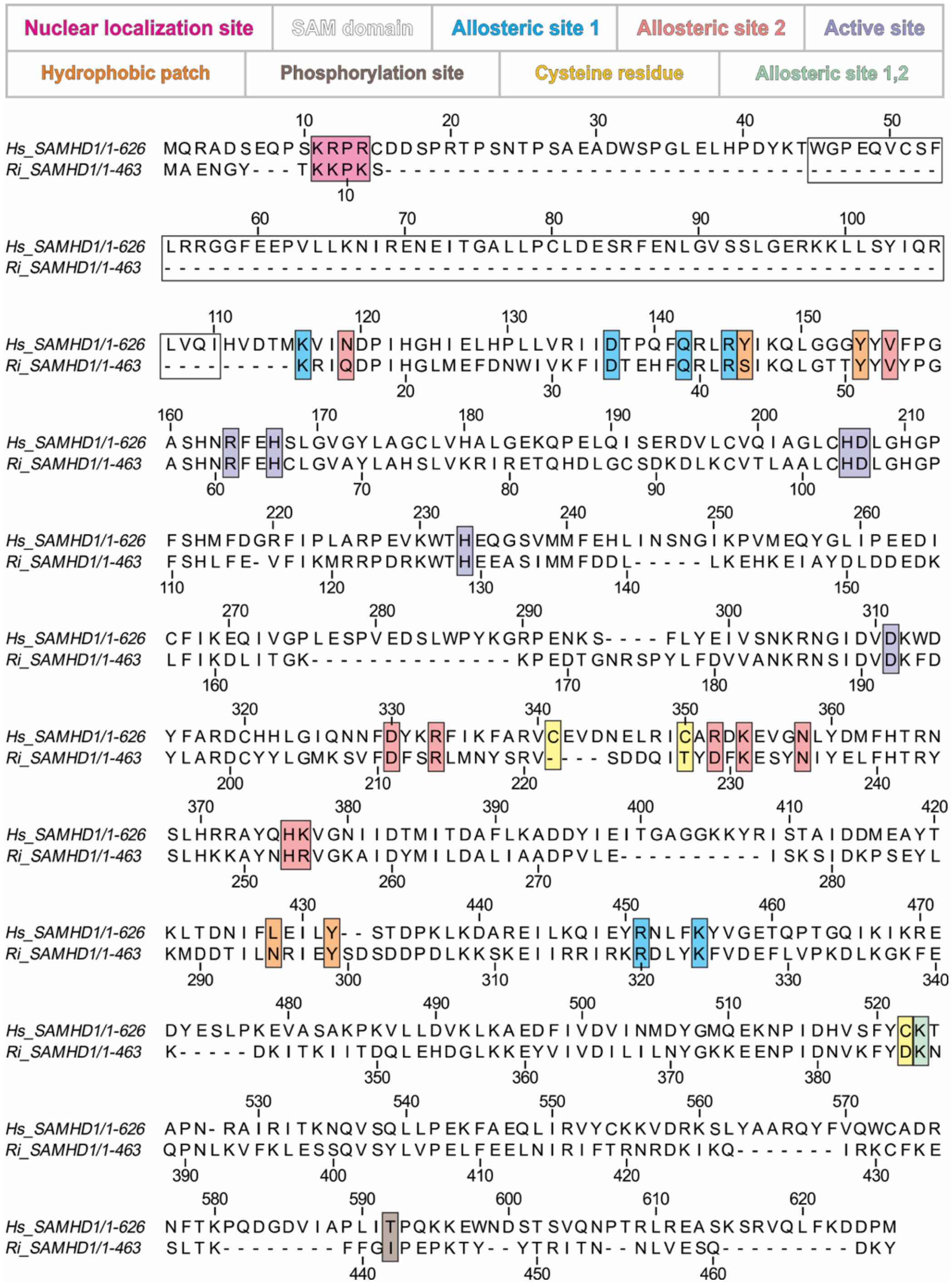

Figure S2

Sequence alignment of *Hs* SAMHD1 and *Ri* SAMHD1. The nuclear localization signal, SAM-domain, the redox-active cysteines (C341,350, and C522S) and the phosphorylation target residue (T592) present in *Hs* SAMHD1 are absent from the sequence of *Ri* SAMHD1. Residues from nuclear localization domain, SAM domain, AL 1, AL 2, and active site are highlighted in magenta, orange, blue, pink, and purple, respectively. The lysine residue (K387 in *Ri* SAMHD1, K523 in *Hs* SAMHD1) appears in both AL1 and AL2 is highlighted in green.

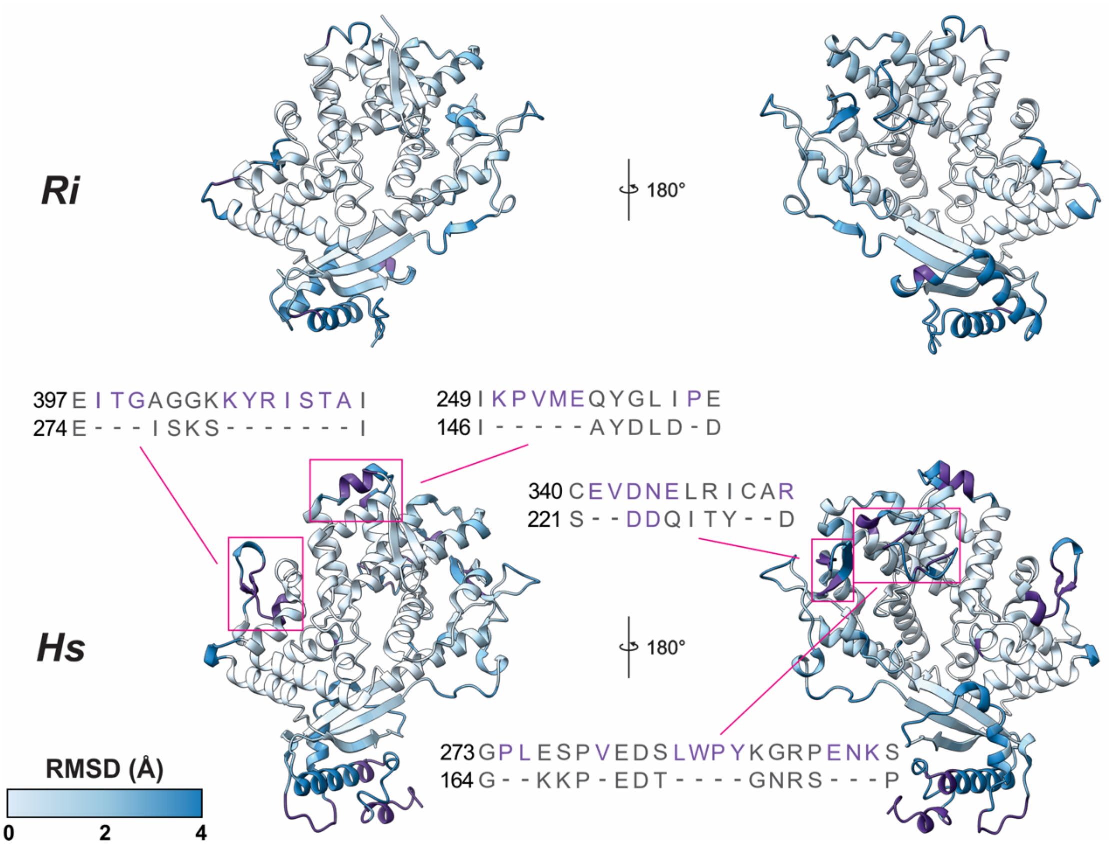

Figure S3

The structures of the subunit B of *Ri* SAMHD1(PDB: 9MR6) and *Hs* SAMHD1 (PDB ID: 7UJN) are colored by the Root-mean-square deviation (RMSD) value mapped onto *Ri* SAMHD1 structure (top) and *Hs* SAMHD1 structure (bottom), using a color scale from white (0 Å) to blue (4 Å). RMSD between 301 pruned atom pairs is 0.981 Å, and RMSD across all 433 pairs is 3.707 Å. Sequences that are not present in the other structure are shown in purple. Three significant differences between the two structures are highlighted in magenta boxes, along with a corresponding sequence comparison (top: *Hs* SAMHD1, bottom: *Ri* SAMHD1) based on the structures.

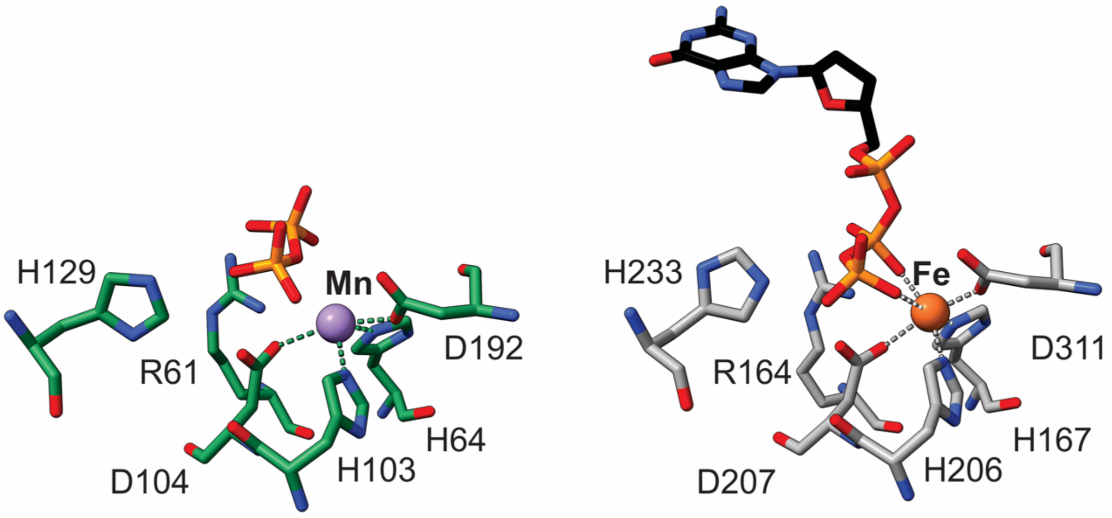

Figure S4

Detailed views of the active sites ~~and allosteric sites~~ of *Ri* SAMHD1 (green, PDB: 9MR6) and *Hs* SAMHD1 (grey, PDB: 5AO0). Bound nucleotide is colored in black. Coordination bonds between metal ions and amino acids are shown as dotted lines. The metal ions Mn^{2+} and Fe^{2+} are represented by purple and orange spheres, respectively.

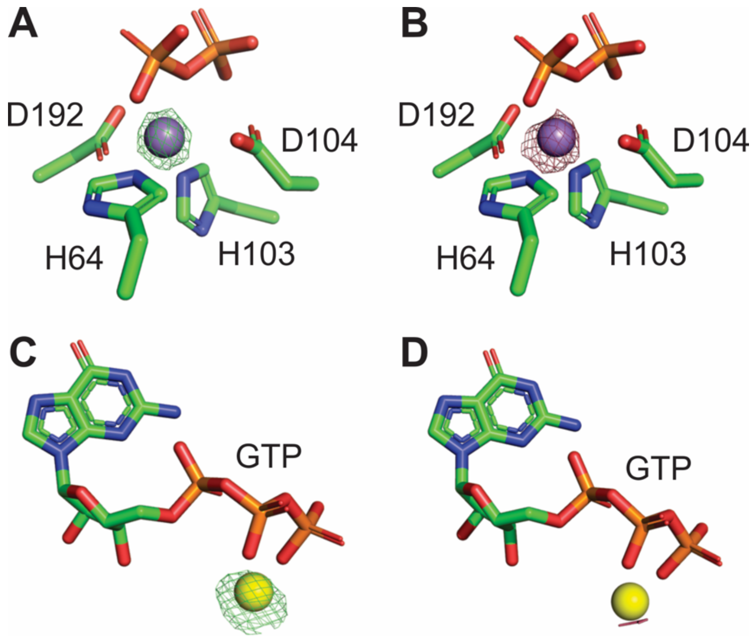

Figure S5.

(A) Omit map (Fo-Fc) at 16σ and (B) anomalous map at 3.5σ of the Manganese ion in chain A coordinated to active site residues D104, H103, H64, D192 and pyrophosphate (C) Omit map (Fo-Fc) at 6.5σ and (D) anomalous map at 1σ of the calcium ion in the allosteric site between chains A and B, coordinated to the β- and γ-phosphate of GTP. The metal ions Mn^{2+} and Co^{2+} are represented by purple and yellow spheres, respectively.

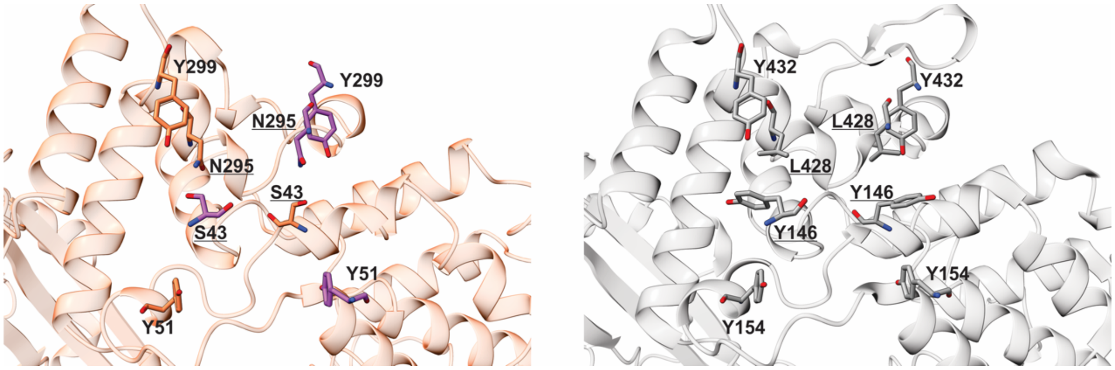

Figure S6

A close-up view of the four hydrophobic residues that stabilized *Hs* SAMHD1 dimer interface (right, PDB: 7UJN) and their coordinating residues in *Ri* SAMHD1 (left, PDB: 9MR6). *Ri* SAMHD1 subunits C and D are colored in purple and orange, respectively.

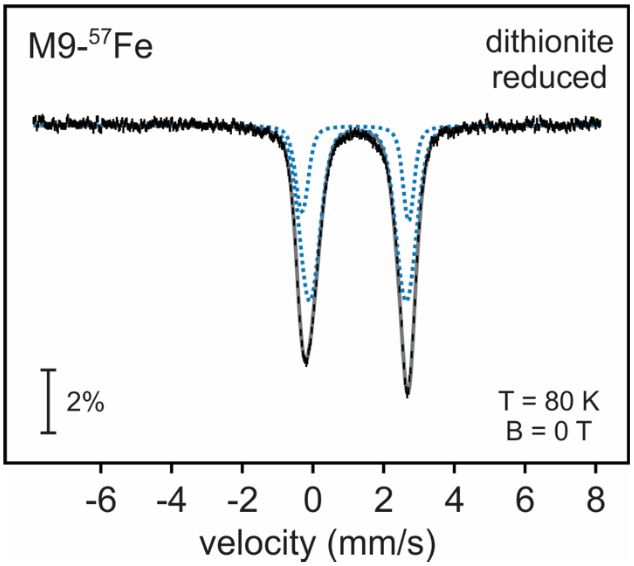

Figure S7.
^{57}Fe Mössbauer spectra of sodium dithionite reduced M9-Fe WT *Ri* SAMHD1 recorded at T = 80 K. Experimental spectra are depicted with black solid bars. The grey solid line corresponds to total fits, the blue dotted lines correspond to the constituent doublets.

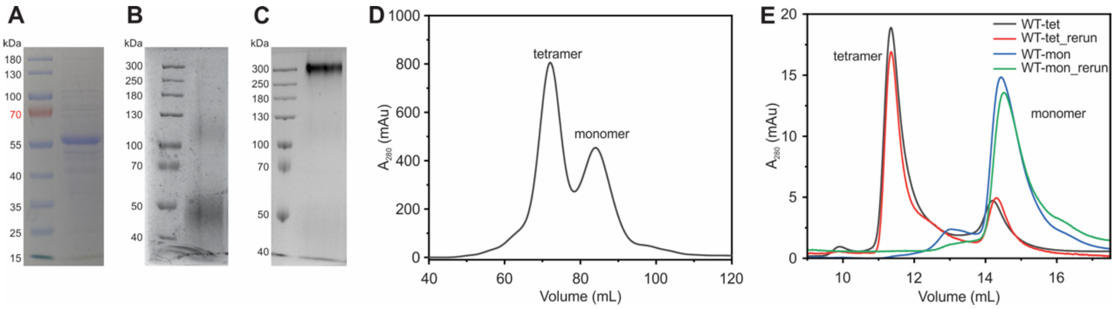

Figure S8.

Purity and oligomeric states of the as-purified *Ri* SAMHD1.

(A) 12% SDS-PAGE of as the purified M9-Fe *Ri* SAMHD1, running at the expected molecular weight of ~57 kDa. (B) Chemical cross-linking gels of 2 μM WT *Ri* SAMHD1 monomer incubated with 20 mM glutaraldehyde. (C) Chemical cross-linking gels of 2 μM WT *Ri* SAMHD1 tetramer incubated with 20 mM glutaraldehyde. (D) Representative preparative SEC of WT *Ri* SAMHD1 (black) demonstrating that the protein elutes at the approximate molecular weights expected for monomer and a tetramer, respectively. (E) Oligomeric state determination of the concentrated fractions of the monomer (blue) and the tetramer (black) *Ri* SAMHD1 using analytical SEC. The representative peaks corresponding to the monomer and tetramer were collected and concentrated, and subsequently subjected to a second round of SEC analysis to interrogate the stability of the respective states (monomer: green, tetramer: red).

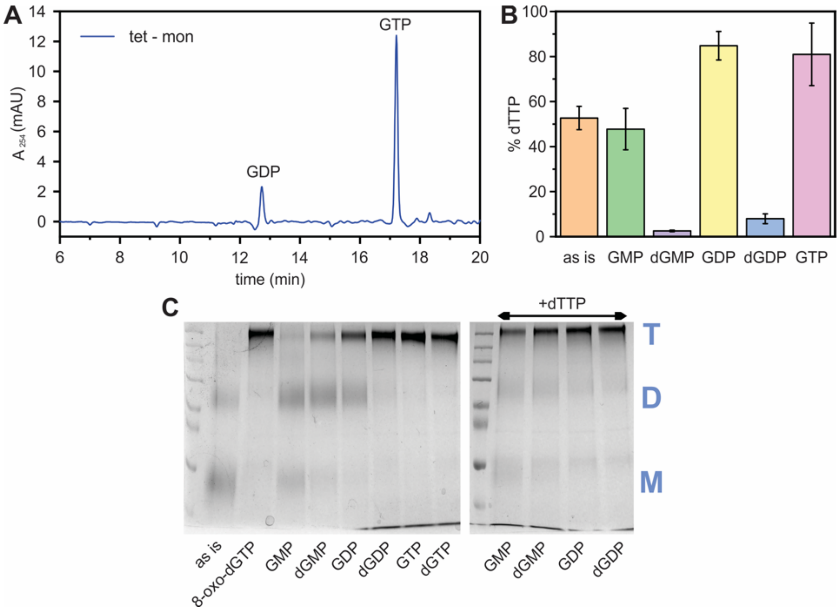

Figure S9.

Analysis of copurifying molecules between as-purified *Ri* SAMHD1 monomer and tetramer.

(A) Comparison of HPLC chromatograms for denatured as-purified M9-Fe *Ri* SAMHD1 monomer and tetramer. (B) End-point assay monitoring dTTP hydrolysis by M9-Fe WT *Ri* SAMHD1 monomer under various 0.5 mM G-based nucleotides adding conditions in the presence of Mn as an activator. Experimental conditions: [*Ri* SAMHD1] = 0.5 μM, [dTTP] = 0.5 mM, [Mn^{2+}] = 1 mM, [NaDT] = 5 mM. Reaction time with the reducing agent NaDT was 20 min, while the end-point assay was quenched after 30 min. (C) Chemical cross-linking gels monitoring oligomerization of WT *Ri* SAMHD1. The WT monomer Ri SAMHD1 was incubated with Mn^{2+} (0.5 mM) and (left) 0.5 mM G-based nucleotides, (right) both dTTP (0.5 mM) and G-based nucleotides (0.5 mM), followed by reaction with 20 mM glutaraldehyde for 15 min. The positions of monomer (M), dimer (D), and tetramer (T) are indicated.

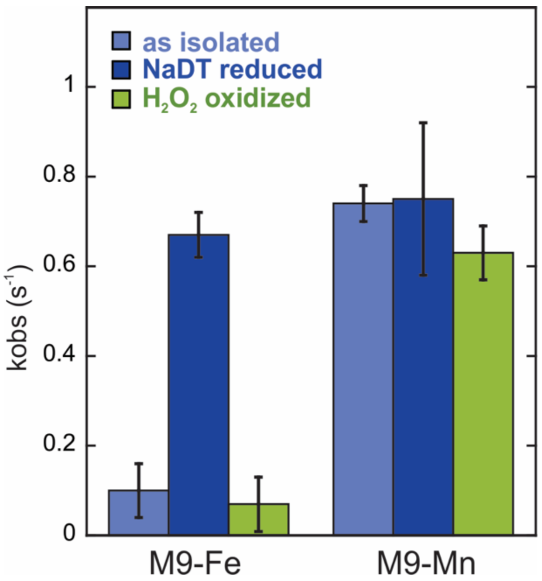

Figure S10.

End-point assay monitoring dGTP hydrolysis by the M9-Mn and M9-Fe WT *Ri* SAMHD1 monomer under different redox conditions in the presence of Mn as an activator under anoxic conditions. Mn cannot detectably replace the metal ions at the active site as the reoxidized M9-Fe *Ri* SAMHD1 is still redox sensitive.

(pale blue) as-purified enzyme, (deep blue) reduced with excess sodium dithionite, (green) reoxidized with excess H_2O_2 after reducing with excess sodium dithionite and addition of Mn. Experimental conditions: [*Ri* SAMHD1] = 0.5 µM, [dGTP] = 0.5 mM, [Mn^{2+}] = 1 mM, [NaDT] = 5 mM, [H_2O_2] = 10 mM. Reaction time with the reducing /oxidizing agents was 15 min, while the end-point assay was quenched after 20 min.

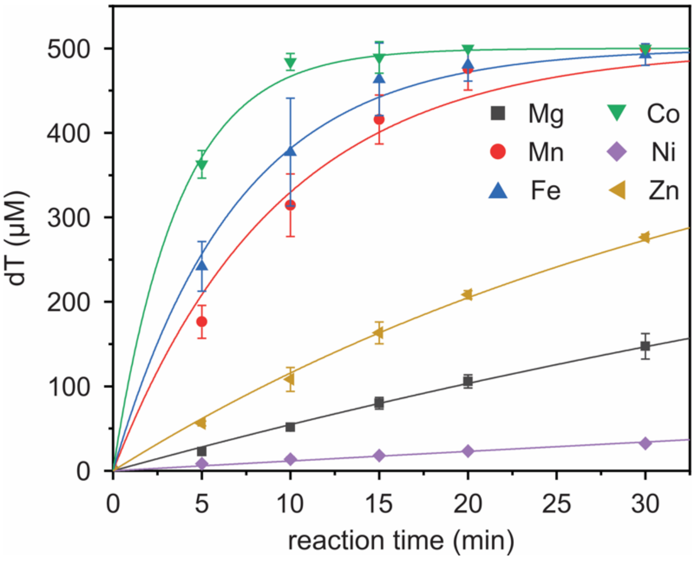

Figure S11.
dTTP hydrolysis by the WT *Ri* SAMHD1 tetramer after addition of 1 mM Mg^{2+} (black), Mn^{2+} (red), Co^{2+} (green), Ni^{2+} (purple), Zn^{2+} (yellow), or 0.5 mM Fe^{2+} (blue). All assays were performed under O_2-free conditions with a final concentration of protein 0.5 μM and 0.5 mM of dTTP.

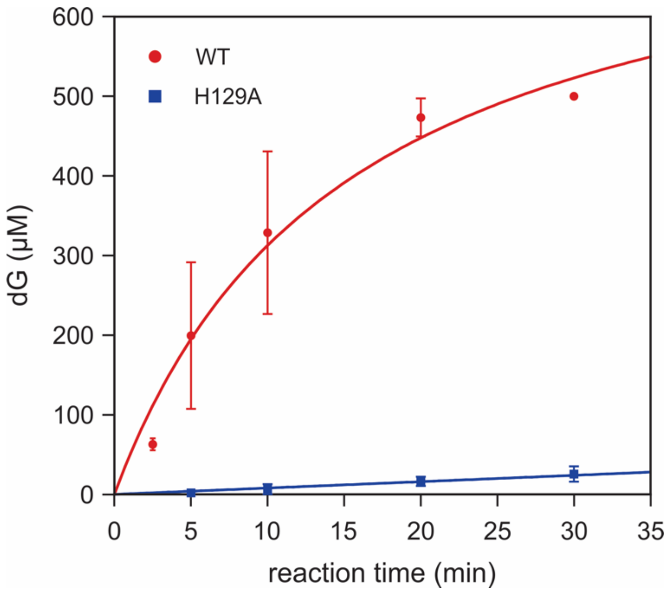

Figure S12.

dGTP hydrolysis by the M9-Mn WT (red) and H129A (blue) *Ri* SAMHD1 tetramer in the presence of Mn as an activator.

Experimental conditions: [*Ri* SAMHD1] = 0.5 µM, [dGTP] = 0.5 mM, [Mn^{2+}] = 1 mM.

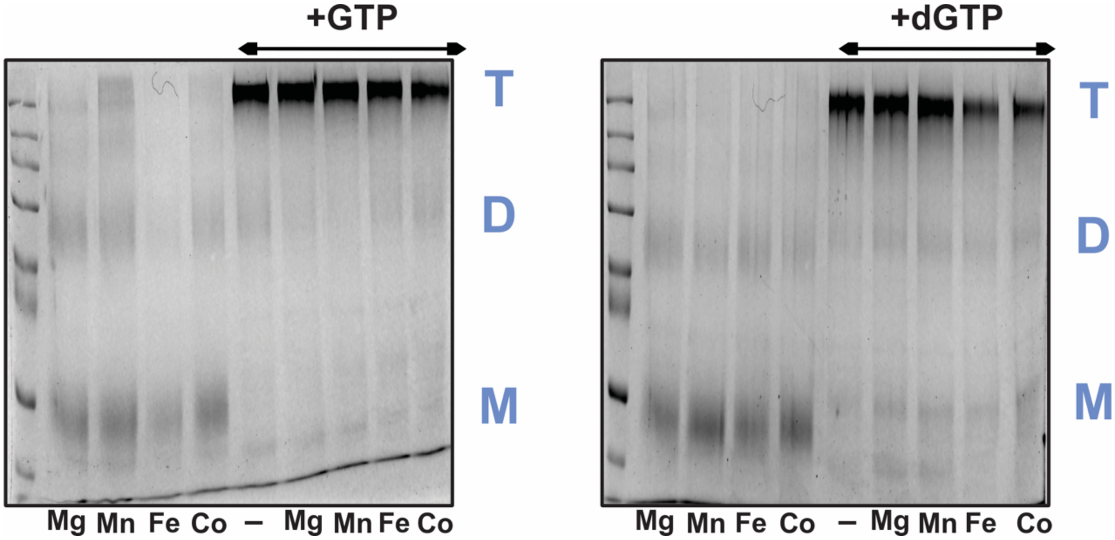

Figure S13.
Chemical cross-linking gels monitoring oligomerization of WT *Ri* SAMHD1.
The WT monomer *Ri* SAMHD1 was incubated with (left) GTP and (right) dGTP (500 µM) and Mg^{2+}, Mn^{2+}, Co^{2+} (1 mM) or Fe^{2+} (0.5 mM) followed by reaction with 20 mM glutaraldehyde for 15 min. The positions of monomer (M), dimer (D), and tetramer (T) are indicated.

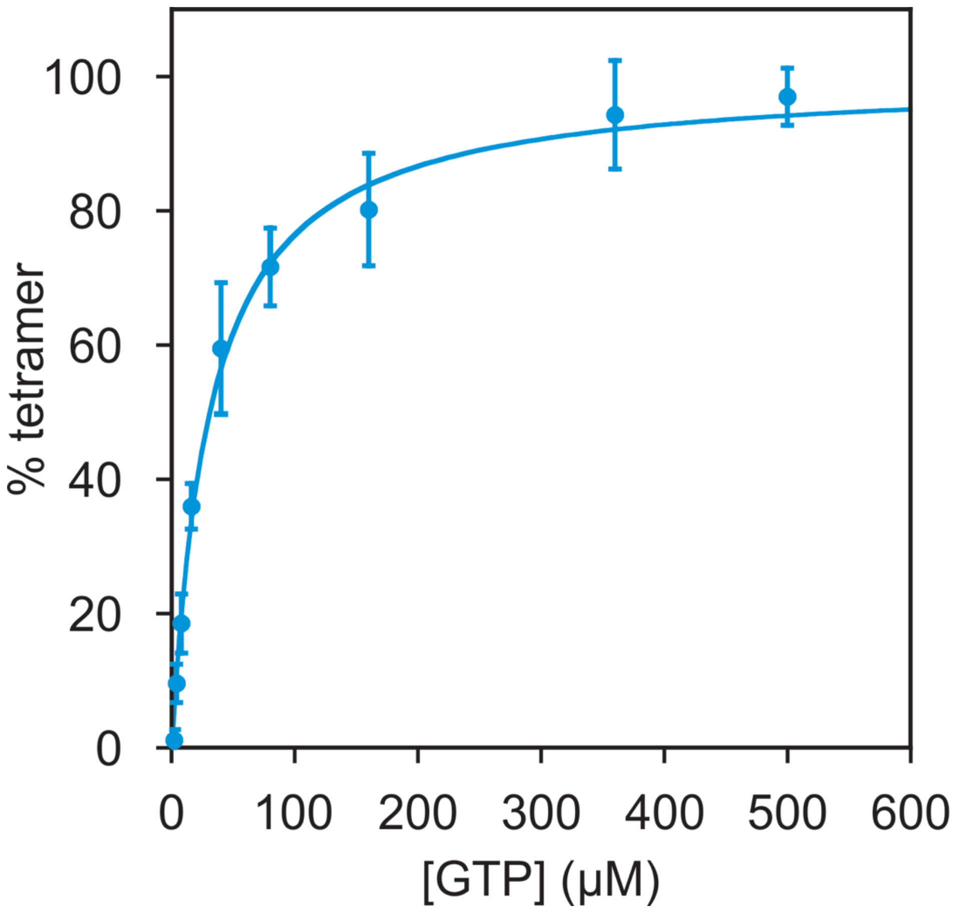

Figure S14.

Tetramerization of the *Ri* SAMHD1 WT monomer as a function of GTP concentration. 2 μM of *Ri* SAMHD1 was incubated with GTP (2 μM to 500 μM) and Mn^{2+} (1 mM) followed by incubation with 20 mM glutaraldehyde for 15 min. The percent formation of tetramer was quantified by estimating the relative intensity of the gel bands using ImageJ.

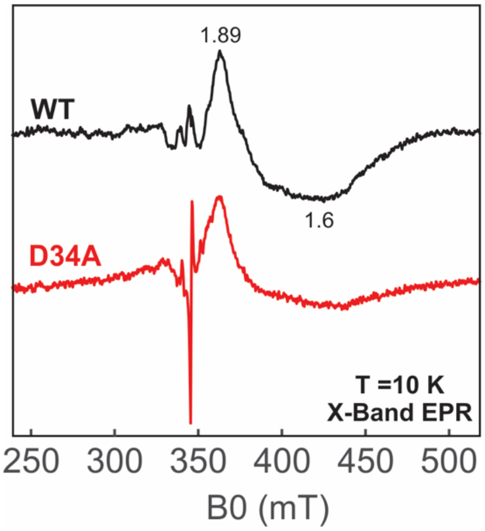

Figure S15.

CW X-Band EPR spectra of the WT and D34A *Ri* SAMHD1.

The WT (black) and D34A *Ri* SAMHD1 (red) were reduced with 2 molar eq sodium ascorbate under O_2-free conditions. Experimental conditions: temperature = 10 K, microwave frequency = 9.38 GHz, microwave power = 2 mW, modulation amplitude = 1 mT.

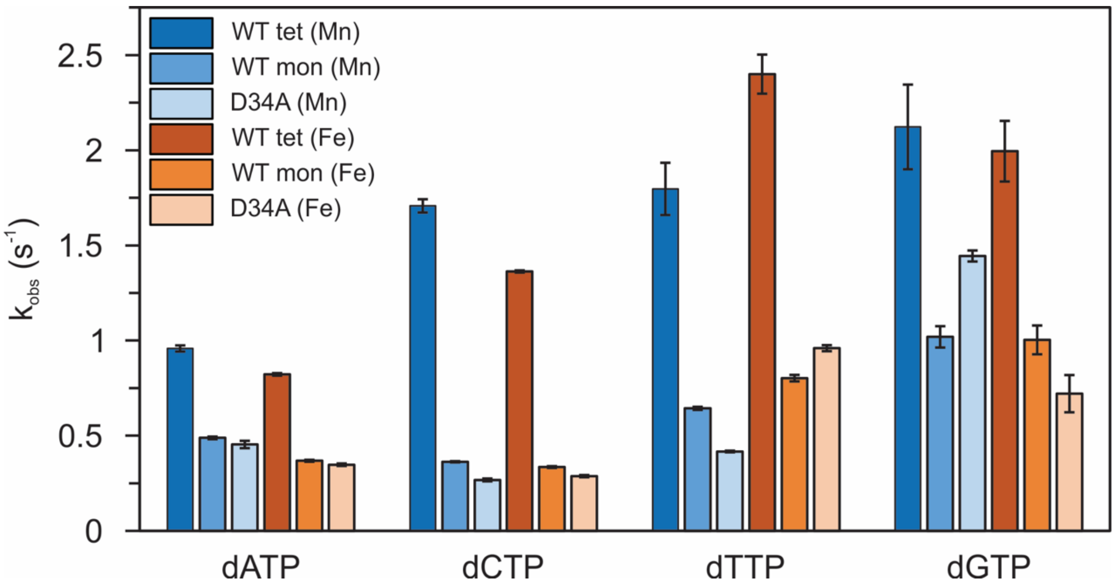

Figure S16.

Endpoint assays monitoring dNTP hydrolysis by WT and D34A *Ri* SAMHD1.

dNTP hydrolysis by the different oligomeric forms of the WT *Ri* SAMHD1 and monomer D34A in the presence of 1 mM Mn^{2+} (blue), or 0.5 mM Fe^{2+} (brown). All assays were performed under O_2-free conditions with a final concentration of protein 0.5 µM and 0.5 mM of dNTP.

Supplemental Tables

Table S1. List of Primers employed in this study.

Ri **SAMHD1 variants**	**Vector**	**Restriction Sites**	**Forward Primer (5' – 3')**	**Reverse Primer (5' – 3')**
**H129A**	pET-28a (+)	Ndel, Xhol	TAAATGGACCGCCGAAGAAGCTAGCATCATGATG	CGATCCGGACGACGCATC
**H103R/D104N**	pET-28a (+)	Ndel, Xhol	GCGGCTTTGCCGCAATCTAGGCCATG	GCCAGGGTAACACACTTCAGG

Table S2. Crystallography data and refinement statistics. Data were collected at NSLS II beamline 17-ID-2. Highest resolution shell is shown in parenthesis (¶).

	***Ri* SAMHD1 (PDB: 9MR6)**
**Data processing**	
Wavelength (Å)	0.9794
Space group	P 1
Cell dimensions	
α, β, γ (°)	84.359, 85.42, 87.035
a, *b*, *c* (Å)	104.58, 111.635, 99.513
Resolution (Å)¶	76.04 - 2.27 (2.31 - 2.27)
Redundancy¶	1.8 (1.7)
Completeness (%)¶	94.6 (84.9)
<I /σI>¶	2.8 (0.5)
R_{merge}¶	0.102 (0.764)
$R_{measure}$¶	0.144 (1.08)
$CC_{1/2}$¶	0.991 (0.383)
Wilson *B*-factor ($Å^2$)	46.43
**Refinement**	
No. reflections¶	89914 (7518)
Resolution (Å)¶	19.8 - 2.27 (2.351 - 2.27)
R_{work} / R_{free}¶	0.2153 (0.3644)/0.2731 (0.3997)
No. atoms	15060
Protein	14751
Mn^{2+}, Ca^{2+}	4, 4
Water	137
B-factors ($Å^2$)	
Protein	65.03
Ligand/ion	61.22
Water	48.71
R.m.s. deviations	
Bond lengths (Å)	0.0009
Bond angles (°)	0.71
Ramachandran analysis	
Favored (%)	96.11
Allowed (%)	3.83
Outliers (%)	0.06

Table S3. Sequence comparison of *Ri* SAMHD1 with human and plant SAMHD1s.

	Alignment Length	Identical Residues	Similar Residues	Percent Identity	Percent Similarity
Hs SAMHD1	530	207	72	39.06	52.64
Os SAMHD1	522	168	87	32.18	48.85
Cs SAMHD1	499	174	91	34.87	53.11

Table S4. Elemental analysis of WT *Ri* SAMHD1 expressed in different media employed in this study as determined by ICP-AES.

Media	Fe (mol/mol protein)	Mn (mol/mol protein)	Ni (mol/mol protein)	Co (mol/mol protein)	Mg (mol/mol protein)	Zn (mol/mol protein)
M9-Fe[a]	0.97 ± 0.31	<0.01	0.21 ± 0.19	<0.01	0.66 ± 0.86	0.29 ± 0.13
M9-Mn	0.26	0.18	0.04	<0.01	0.10	0.39

[a] Average result from four independent protein preparations

Table S5. Results of fit for GTP regulation

	k_{cat} (s^{-1})	K_M (mM)	K_I (mM)
dATP	5.26 ± 1.43	0.53 ± 0.29	0.05 ± 0.02
dCTP	4.43 ± 0.63	0.65 ± 0.19	0.06 ± 0.01
dTTP	2.73 ± 0.41	0.18 ± 0.08	0.39 ± 0.11

References

1. Ignesti, G., *Equations of substrate-inhibition kinetics applied to pig kidney diamine oxidase (DAO, E.C. 1.4.3.6)*. J Enzyme Inhib Med Chem, 2003. **18**(6): p. 463-73.
